# Single Cell Transcriptomic and Chromatin Profiles Suggest Layer Vb Is the Only Layer With Shared Excitatory Cell Types in the Medial and Lateral Entorhinal Cortex

**DOI:** 10.3389/fncir.2021.806154

**Published:** 2022-01-26

**Authors:** Stefan Blankvoort, Lene Christin Olsen, Clifford G. Kentros

**Affiliations:** ^1^Kavli Institute for Systems Neuroscience and Centre for Neural Computation, NTNU, Trondheim, Norway; ^2^Department of Clinical and Molecular Medicine, Norwegian University of Science and Technology, Trondheim, Norway; ^3^BioCore Bioinformatics Core Facility, NTNU, Trondheim, Norway; ^4^Department of Neurology, St. Olavs Hospital, Trondheim, Norway

**Keywords:** cell types, ATAC-seq, transcriptomics, entorhinal cortex, enhancers, chromatin, memory, consolidation

## Abstract

All brain functionality arises from the activity in neural circuits in different anatomical regions. These regions contain different circuits comprising unique cell types. An integral part to understanding neural circuits is a full census of the constituent parts, i.e., the neural cell types. This census can be based on different characteristics. Previously combinations of morphology and physiology, gene expression, and chromatin accessibility have been used in various cortical and subcortical regions. This has given an extensive yet incomplete overview of neural cell types. However, these techniques have not been applied to all brain regions. Here we apply single cell analysis of accessible chromatin on two similar but different cortical regions, the medial and the lateral entorhinal cortices. Even though these two regions are anatomically similar, their intrinsic and extrinsic connectivity are different. In 4,136 cells we identify 20 different clusters representing different cell types. As expected, excitatory cells show regionally specific clusters, whereas inhibitory neurons are shared between regions. We find that several deep layer excitatory neuronal cell types as defined by chromatin profile are also shared between the two different regions. Integration with a larger scRNA-seq dataset maintains this shared characteristic for cells in Layer Vb. Interestingly, this layer contains three clusters, two specific to either subregion and one shared between the two. These clusters can be putatively associated with particular functional and anatomical cell types found in this layer. This information is a step forwards into elucidating the cell types within the entorhinal circuit and by extension its functional underpinnings.

## Introduction

Cortical brain regions are diverse in their functionality, the cell types they contain, and the intrinsic as well as extrinsic connectivity (Douglas and Martin, 2007; Harris and Shepherd, 2015; Economo et al., 2018). Previous work has defined cell types based on various criteria. These criteria include morphology, connectivity, physiology, and receptive field (Zeng and Sanes, 2017; Luo et al., 2018; Yuste et al., 2020). Additionally, differential gene expression aligns with these characteristics and can by itself also be used as a criterium for different cell types (Zeisel et al., 2015; Saunders et al., 2018; Tasic et al., 2018). Echoing previous observations based on the former criteria, using single cell transcriptomics Tasic et al. (2018) found that cortical regions are diverse with regards to excitatory cell types rather than inhibitory cell types. Furthermore, in the particular case of the entorhinal cortex (EC), Ramsden et al. (2015) found that gene expression in the two subdivisions (lateral and medial, LEC and MEC respectively) is differential predominantly in the superficial layers.

The two subdivisions of the entorhinal cortex are of particular interest because of their similarity in circuitry and anatomical cell types but diversity in functionality and developmental origin (Hafting et al., 2005; Hargreaves et al., 2005; Witter et al., 2017; Tsao et al., 2018). For example, the two regions are similar in their efferent connectivity toward the hippocampus and the presence of *Reelin*+ excitatory cell types in Layer II (LII) (Witter et al., 2017). But they are different in their functional cell types (Hafting et al., 2005; Tsao et al., 2018) and their afferent connectivity (Burwell and Amaral, 1998). The entorhinal cortex interfaces between the hippocampus and the neocortex (Cappaert and Witter, 2015). The relatively understudied deep layers (Va, Vb, and VI) provide efferent connectivity to neocortical regions. They in themselves comprise a complex microcircuit, which has both similarities and differences between the two EC subregions (Cappaert and Witter, 2015; Surmeli et al., 2015; Ohara et al., 2018, 2021).

Integration of the information on cell types, connectivity and functionality gives us a detailed and expansive circuit model of the subregions. However, it is still partially unclear which particular cell types and layers contribute to the similarities and differences between the subregions. Here we make use of single cell Assay for Transposase-Accessible Chromatin using sequencing (scATAC-seq), which allows the exploration of a taxonomy of cell types based on chromatin accessibility (Buenrostro et al., 2013; Cusanovich et al., 2018; Fang et al., 2021). We combine this data with previously published scRNA-seq data to corroborate and refine our findings.

We find all major cell types expected in the EC, representing non-neurons, groups of interneurons and layer specific excitatory neurons. As expected, the non-neuron and inhibitory cell types are equally represented in the LEC and the MEC. In contrast, the excitatory cells in the superficial layers cluster to either the LEC or MEC. Interestingly, several types of the excitatory cells in the deep layers are found both equally represented in LEC and MEC or specific to either region. Based on chromatin profiles, each of the three deep layers has a shared aspect, but Layer Vb (LVb) contains additional regionally specific cell types. Investigation of motifs present in the open chromatin suggests activity of different transcription factors between the shared and unique LVb cell types. Transcriptomic data corroborates this for the LVb cell types. This finding refines our view of the cell types present in LVb of the LEC and MEC, and how these two regions correspond to one another.

## Methods

### Microdissection

Tissues were taken from two (both male) P56 C57B6/J mice from the Jackson laboratory (Stock No: 000664). The mice were deeply anesthetized, decapitated and the brain was removed. The brains were kept in ice cold hibernate-A medium (Thermo Fisher, A1247501) until microdissection. After sectioning (horizontal, 500 μm sections, on a Leica VT 1000 S vibratome) medial and lateral EC were microdissected out from all sections on the dorsal-ventral axis. Conservative cuts were made while watching the tissue through a dissection microscope with transmitted and reflected white light (Zeiss Discovery V8 stereomicroscope) along the borders between the subdivisions of the EC and along borders with other cortical regions applying architectonic criteria (Jones and Witter, 2007; Witter, 2011; Boccara et al., 2015; O'Reilly et al., 2015; Sugar and Witter, 2016) to unstained tissue. All dissections avoided border regions, i.e., were taken centered in the identified cortical area. In horizontal sections, MEC is easily recognized by the marked shape of the cortex, the prominent white, opaque lamina dissecans and the radial organization of the layers deep to the latter. Layer II neurons are large spherical neurons, which differ markedly in level of opacity from those in layer III. The medial border between MEC and parasubiculum is characterized by the loss of the differentiation between layers II and III, and the border with the laterally adjacent postrhinal cortex is characterized by the loss of the large spherical neurons in layer II. We only sampled the more dorsal and central portions of MEC. LEC shares the large layer II neurons with MEC, but the radial organization in layer V is absent. The anterior and dorsal border of LEC with the perirhinal cortex is characterized by the abrupt disappearance of the large layer II neurons.

After microdissection, the tissue was flash frozen in liquid nitrogen and stored at −80°C. The mice were kept on a 12-h light/12-h dark schedule in a humidity and temperature-controlled environment. Experiments were performed in accordance with the Norwegian Animal Welfare Act and the European Convention for the Protection of Vertebrate Animals used for Experimental and Other Scientific Purposes.

### scATAC-Seq

For scATAC-seq the 10X genomics protocols were followed. Briefly, the cells were lysed in a lysis buffer (10 mM Tris-HCl, pH 7.4, 10 mM NaCl, 3 mM MgCl_2_, 0.1% Tween-20, 0.1% Nonidet P40 Substitute, 0.01% Digitonin, 1% BSA). After lysis, the cell suspension was diluted with wash buffer (10 mM Tris-HCl, pH 7.4, 10 mM NaCl, 3 mM MgCl_2_, 0.1% Tween-20, 1% BSA) and filtered by two steps of straining (Bel-Art Flowmi cell strainer, 70 μm H13680–0070, 40 μm H13680–0040). Cells were spun down at 500rcf for 5 min (4°C). After this the cells underwent a final filtration step through a 40 μm strainer (Bel-Art Flowmi cell strainer, H13680–0040). Nuclei concentration was determined by staining the material with a LIVE/DEAD Viability/Cytotoxicity Kit (Thermo Fisher L3224) and automatic counting on a Countess II FL (Thermo Fisher). After this the cells were immediately carried on to prepare for partitioning and barcoding on a chromium controller. Sequencing was done on an Illumina NS500. Partitioning to sequencing was done by the Genomics Core Facility at the St. Olavs hospital, Trondheim.

### Analysis of scATAC Data

For the analysis of scATAC data, the Snaptools/SnapATAC pipeline (Fang et al., 2021). Briefly, FastQ files were aligned to mm10 and resulting, position sorted BAM files were used as input for Snaptools with binsizes of 5 kbp. The resulting file at this point contains sessions with a header, metadata, fragment data and a cell-by-bin accessibility matrix. Subsequently, the snapfiles were loaded into the SnapATAC pipeline. Cells were selected based on unique molecular identifiers (UMI) and promoter ratio, and genomic bins covering ENCODE blacklist regions, non-canonical chromosomes and invariant features were removed. To cluster the cells, the top eigen dimensions of the cell-by-bin matrix representing relevant signal were selected (in this case 22) and a KNN graph was based on this. Clusters were determined by Louvain community detection. After this, UMAP dimensionality reduction was run. Typically, gene accessibility was projected on this graph to identify cell types corresponding to the cluster. Clusters were hierarchically sorted based on pooled genomic signal.

### Finding and Analyzing Differentially Active Regions

First, we generated pseudo bulk signals for each cluster and called peaks on these with MACS2 (options “–nomodel –shift 100 –ext 200 –qval 5e-2 -B –SPMR”) (Zhang et al., 2008). After unification, this yielded a list of 260,218 peaks. We then identified differentially active regions (DARs) for each cluster using FindDAR in the SnapATAC pipeline. We selected the peaks that had an FDR <0.05 and logFC>0. For those clusters with fewer than 1,000 peaks, we selected the top 1,000 peaks (logFC>0, sorted on most significant FDR) for each cluster to maintain sufficient numbers (Supplementary Table 1). We ran Homer (Heinz et al., 2010) known motif search (findMotifsGenome.pl) on DARs of each individual cluster. Additionally, we ran Homer on contrasted unified lists of DARs from multiple clusters (Quinlan and Hall, 2010) (Supplementary Data Sheet 1). This to more generally explore motifs we used these contrasts: excitatory neurons (clusters 1, 2, 3, 7, 8, 10, 14, 15, 16, 19) vs. inhibitory neurons (clusters 4, 5, 13, 20), excitatory superficial neurons of MEC vs. LEC (respectively clusters 3, 7, 8 and 10, 15, 19), excitatory neurons of superficial vs. deep layers (respectively clusters 3, 7, 8, 10, 15, 19, and 1, 2, 14, 16, 17).

### Analysis of scRNAseq Data

The scRNAseq data was analyzed by the Seurat pipeline (Hao et al., 2021). Unique molecular identified (UMI) count matrices for the entorhinal cortex data (Yao et al., 2021) were downloaded from the NeMO archive. After preprocessing (based on numbers of features and percentage of mitochondrial cells) and normalization, the top 2,000 highly variable features were identified for further analysis. Then the data was scaled, and the top 44 eigen dimensions were selected based on differentially expressed genes. Clusters were identified within these top eigen dimensions and UMAP dimensionality reduction was applied. Cell types were identified based on differentially expressed genes. Hierarchical clustering of clusters was done based on average gene expression scores of the 2,000 most variable genes. DE genes were determined using the Wilcoxon rank-sum test, both when determining DE genes of all clusters and when determining DE genes when contrasting selected clusters.

### Mapping Transcriptomic Cell Type on Epigenomic Cell Type

To compare the two incongruent types of data, first a gene matrix with the 2,000 most highly variable genes was added to the SnapATAC file. This was converted to a Seurat object, and anchors were found to harmonize the dataset with the transcriptomic dataset (Stuart et al., 2019). Based on this, a prediction was made based on the distance in CCA (canonical correlation analysis) space of both datasets (gene accessibility for scATAC-seq and gene expression for scRNA-seq).

## Results

### Clustering and Identification of scATAC-Seq Cells

**Figure 1:**
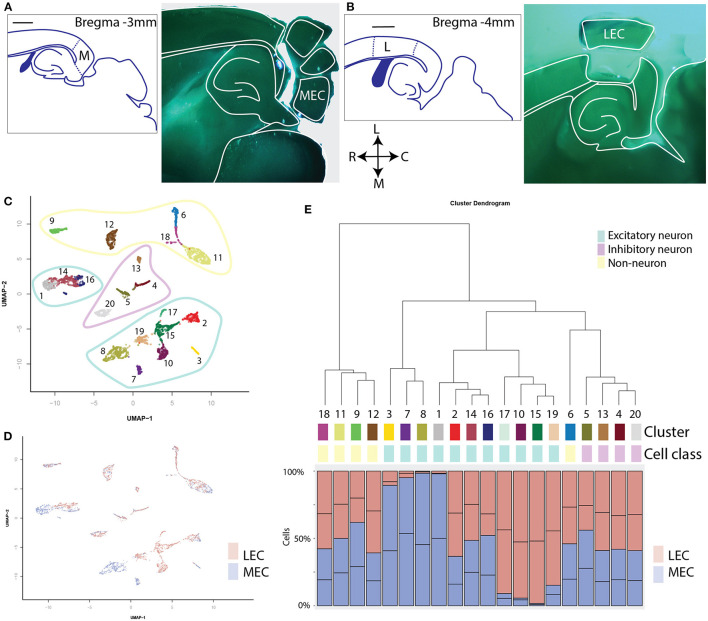
Clustering of all single cells reveals shared and sample-enriched clusters of cells. **(A)** Left: schematic reference of the tissue, scale bar is 1 mm, M indicates the MEC. Right: Example of microdissection of subregions of the EC and approximate level in an anatomical atlas. This section was cut horizontally to optimally allow dissection of both subregions of the EC. **(B)** Schematic reference and example of microdissection of the lateral subregion of the EC. L indicates the LEC. **(C)** UMAP projection of all cells profiled with scATAC sequencing. Coloring and labeling indicate cluster numbers of scATAC data used in the rest of this publication. The outlines indicate three different classes of cells: non-neurons (yellow), inhibitory neurons (magenta), and excitatory neurons (cyan). **(D)** UMAP projection labeled by sample. Note the existence of both mixed clusters and clusters consisting primarily of either MEC or LEC cells. **(E)** Hierarchical dendrogram of the clusters of scATAC cells with quantification of the contribution of each sample to each cluster. Contributions are given in percentages. Cluster numbers, cell class, and colors correspond with those in **(C)**.

We applied scATAC-seq on 2 replicates each of the MEC and the LEC (Figures 1A,B). After filtering based on unique reads per cell and the percentage of reads present in known promoters, we collected 4,136 single nuclei (Supplementary Figure 1). We clustered based on the top 22 principal components of genome-wide, binned chromatin accessibility, and found 20 separate clusters (Figure 1C; Supplementary Figure 2A). Clustering was not driven by any technical metrics such as read depth, duplicated reads or fraction of reads in peaks (Supplementary Figures 2B–E). Each cluster contained between 55 and 472 individual member cells (Supplementary Figure 2F). Hierarchical clustering showed 5 major clades (Figure 1E). Two of these have an approximately equally many LEC and MEC members in each cluster, two have clusters that are made up of only MEC or only LEC members (Figures 1D,E). And a final clade is a mixture between these two types, with three mixed clusters and one MEC enriched cluster. Clusters that are shared between regions and others that are unique to a particular region is reminiscent of previous observations of shared and unique cell types in different cortical regions. To identify the cell types in these clusters we need to put them in a context we understand by annotating them with gene expression.

To investigate the identity of the clusters we labeled member cells with a pseudo-gene expression score, based on the accessibility of the gene (coordinates from Gencode VM16). Genes were selected from known marker genes (Zeisel et al., 2015). This identified clusters of microglia (Supplementary Figure 3, *C1qc*), oligodendrocytes (*Hapln2*), endothelial cells (*Flt2*), astrocytes (*Lcat*). Additionally, cluster 12 possibly contains ependymal, indicated by the expression of the genes *Ccdc153* and *Dynlrb2* (Supplementary Figures 3E,F). Pan-neuronal gene Snap25 was used to identify all neuronal clusters (Supplementary Figure 3G). Within this subset, clusters 1, 2, 3, 7, 8, 10, 14, 15, 16, 17, and 19 are excitatory (Supplementary Figure 3I, *Slc17a7a*), while clusters 4, 5, 13, and 20 are inhibitory (Supplementary Figure 3H, *Pnoc*). Information on the accessibility of chromatin allows us to investigate enrichment of motifs in differentially accessible regions (DARs) of open chromatin (Supplementary Figure 4). We found that the inhibitory neurons were enriched for motifs *Bhlhe22, Meox2*, and *Thrb*, whereas excitatory neurons were enriched for *E2A, Atoh1*, and *Egr2* (Supplementary Figure 3J).

**Figure 2:**
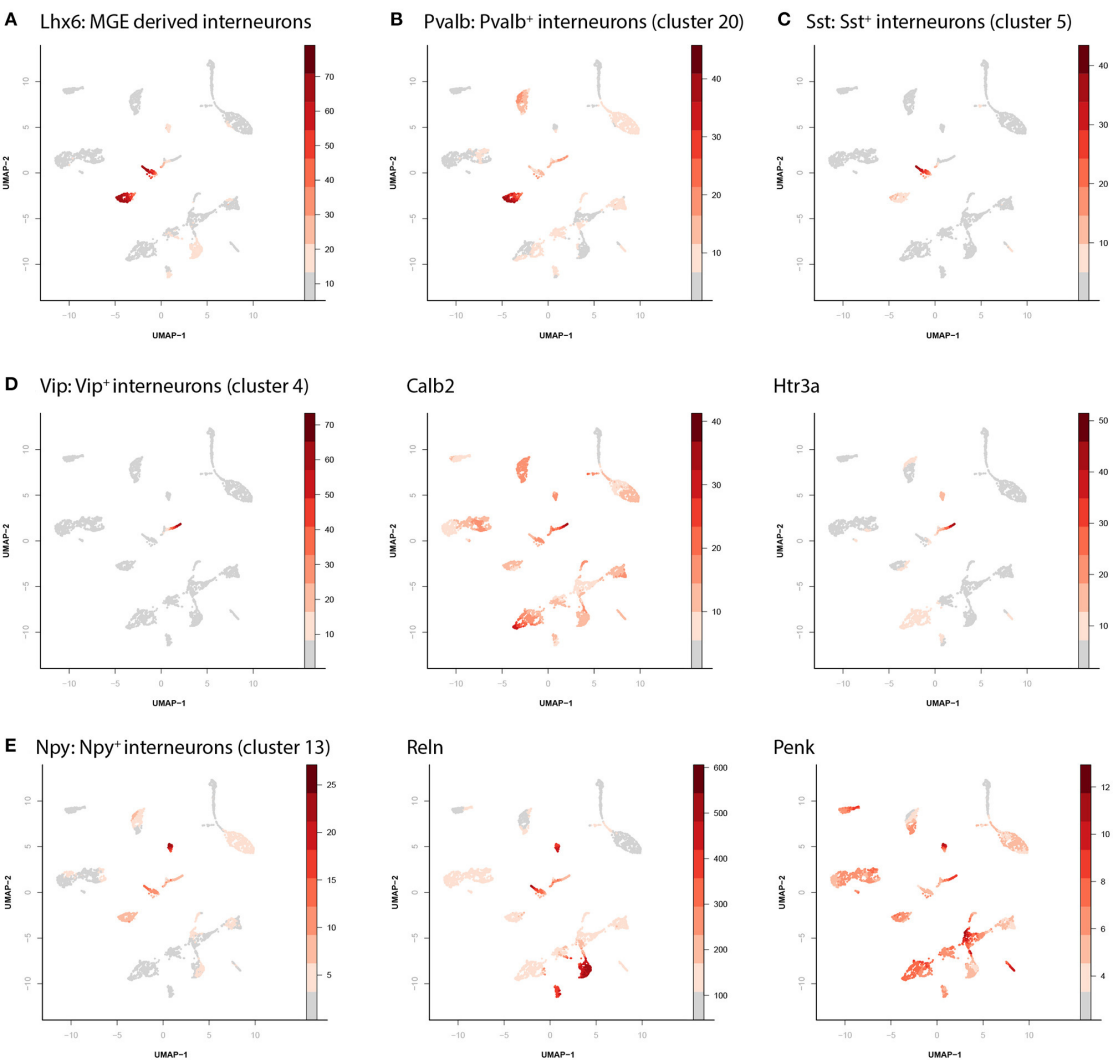
Identification of interneuron cell types. **(A)** There are four different clusters containing inhibitory neurons. Different marker genes can identify the identity of each of these. Here the relative accessibility score of *Lhx6* labels the MGE derived interneurons. **(B)** Accessibility of *parvalbumin* labels parvalbumin positive interneurons (cluster 20). **(C)** Accessibility of *somatostatin* labels somatostatin positive interneurons (cluster 5). **(D)** Accessibility of *vasoactive intestinal peptide* (*VIP*), *calbindin*, and *5-Hydroxytryptamine Receptor 3A* (*Htr3a*) labels the *VIP*+ interneurons (cluster 4). **(E)** Accessibility of *neuropeptide Y* (*NPY*), *Reelin*, and *proenkephalin* (*Penk*) labels the *NPY*+ interneurons (cluster 13).

The clusters with inhibitory cells can be identified as 4 distinct, previously physiologically and morphologically described, biologically relevant subtypes. Cluster 5 and 20 both express *Lhx6*, a marker for medial ganglionic eminence derived neurons (Figure 2A). During development the medial ganglion gives rise to two types of cortical interneurons (Bandler et al., 2017; Lim et al., 2018). Those expressing *Parvalbumin* (*Pvalb*, cluster 20, Figure 2B) and those expressing *Somatostatin* (*Sst*, cluster 5, Figure 2C). The apparently homogenous cells (on a chromatin accessibility level) in cluster 20 can still be subdivided into three different anatomical cell types (the chandelier, basket and axo-axonic cells). Similarly, the cells contained in cluster 5 (*Sst* expressing) can be separated into martinotti cells and non-martinotti cells based on morphology, although this is not apparent from our data. In addition to these two clusters, we identified a cluster of *Vip/Calb2/Htr3a* expressing interneurons (Figure 2D, cluster 4) and a cluster of *Npy/Reln/Penk* expressing interneurons (Figure 2E, cluster 13).

**Figure 3:**
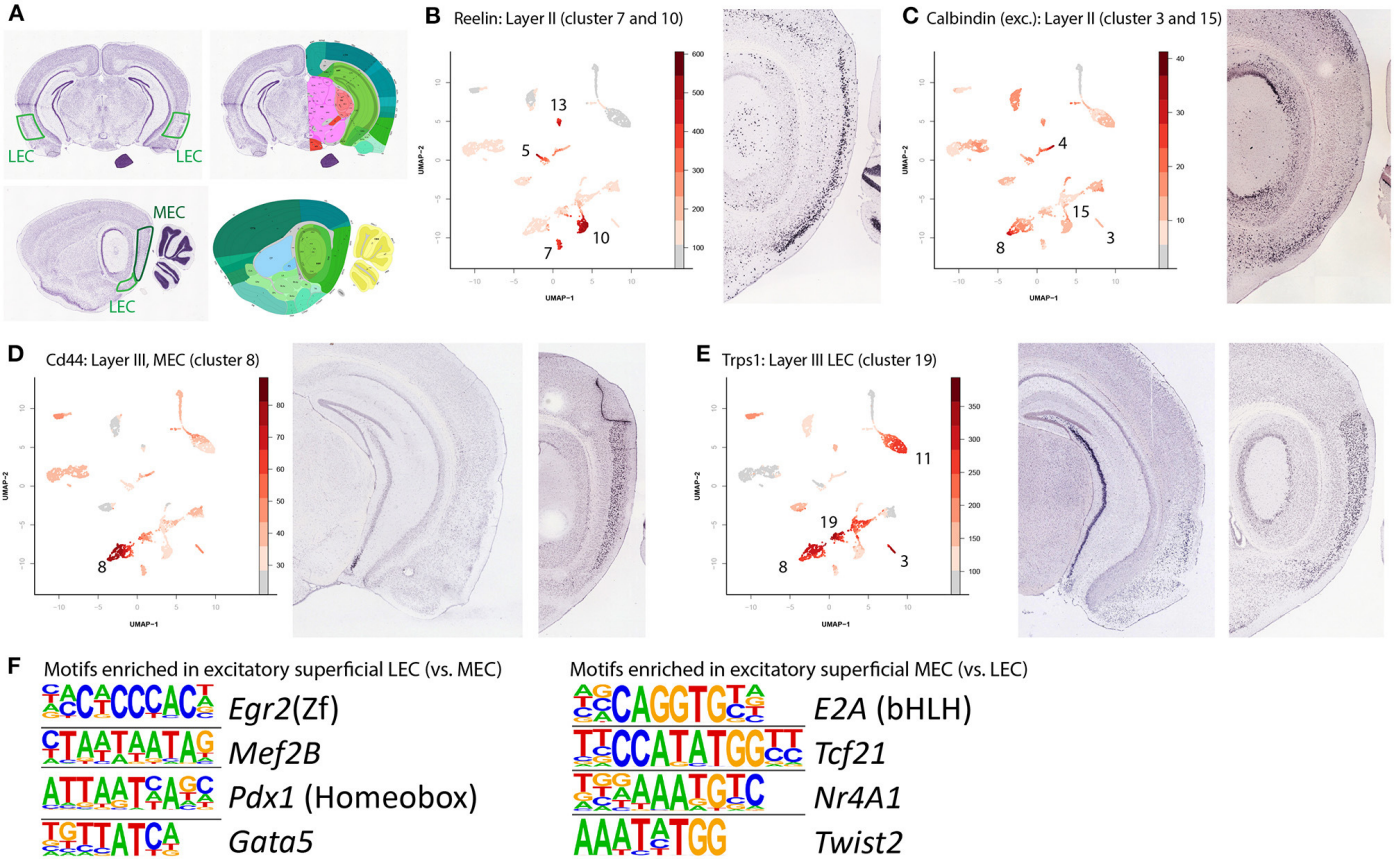
Identification of superficial layer excitatory neurons and enriched motifs. **(A)** Nissl stained and reference images of a mouse brain. Used to identify gene expression in the two different subregions of the EC. Left panels are coronal (3.1 mm posterior to bregma) right panels are sagittal (3.3 mm lateral from the midline). Image taken from Lein et al. (2007). **(B)** Superficial neurons of the entorhinal cortex can be grouped into three broad categories: the *reelin* and *calbindin* expressing LII cells and the layer III cells. Here the *Reelin* expressing LII cells (clusters 7 and 10 for MEC and LEC respectively) are labeled by accessibility of the *Reelin* gene. Note that this gene is also expressed in various inhibitory neuron cell types. **(C)** Accessibility of the *Calbindin* gene labels the *Calbindin* positive cell types (clusters 3 and 15, for MEC and LEC respectively). Similar to *Reelin, Calbindin* is also expressed in various inhibitory cell types. **(D)** Accessibility of *CD44* labels MEC LIII cells (cluster 8). **(E)** Accessibility of *transcriptional repressor GATA binding 1* (*Trps1*) but absence of accessibility of *CD44* labels LEC LIII neurons (cluster 19). **(F)** Detection motifs enriched in excitatory, superficial cells of either the LEC or the MEC. The left sides show the enriched motifs, while the name behind denotes the corresponding best matching transcription factor.

The clusters with excitatory neuronal cell types can be assigned to individual layers based on layer specific genes. To select relevant genes, we used data from Ramsden et al. (2015), which is specified to the entorhinal cortex. All genes were visually confirmed from data in the Allen brain atlas (Lein et al., 2007) (Figure 3A). Using genes with layer specificity they found we were able to find the identity of each cluster of excitatory neurons.

We found six clusters to be specific for the superficial layers of the EC, with each one either LEC or MEC specific. Clusters 7 and 10 both express *Reelin* (*Reln*, Figure 3B, cluster 7 is MEC specific, cluster 10 is LEC specific). In excitatory cells this gene is specifically expressed in LII, Dentate Gyrus projecting neurons. In the adult cortex, *Reelin* is expressed primarily in inhibitory interneurons, the prominent expression of *Reelin* in excitatory LII cells of the EC seems to be an exception (Pesold et al., 1998). The other excitatory cell type in LII is specified by expression of *Wfs1* and *Calbindin*, we find this cell type in clusters 3 (MEC specific) and 15 (LEC specific, Figure 3C). In rodents, these cells are anatomically grouped in “islands.” Like *Reelin*, in most of the adult cortex, *calbindin* is primarily associated with interneurons, but the EC has a population of excitatory *calbindin* expressing cells in LII (Markram et al., 2004; Ray et al., 2014). LIII excitatory cells were found in clusters 8 and 19 (Figures 3D,E), identified with the genes *Cd44* and *Trps1* respectively. Here also, we could detect DARs and find motif enrichment for individual clusters and contrasted clusters (Supplementary Table 1). When contrasting LEC and MEC superficial excitatory neurons, we find that MEC is enriched for *E2A, Tcf21, Nr4a1*, and *Twist2*, whereas LEC is enriched for *Egr2, Mef2B, Pdx1*, and *Gata5* (Figure 3F). Each region accounts for one of the top enriched motifs found in the DARs with high accessibility in excitatory neurons.

**Figure 4:**
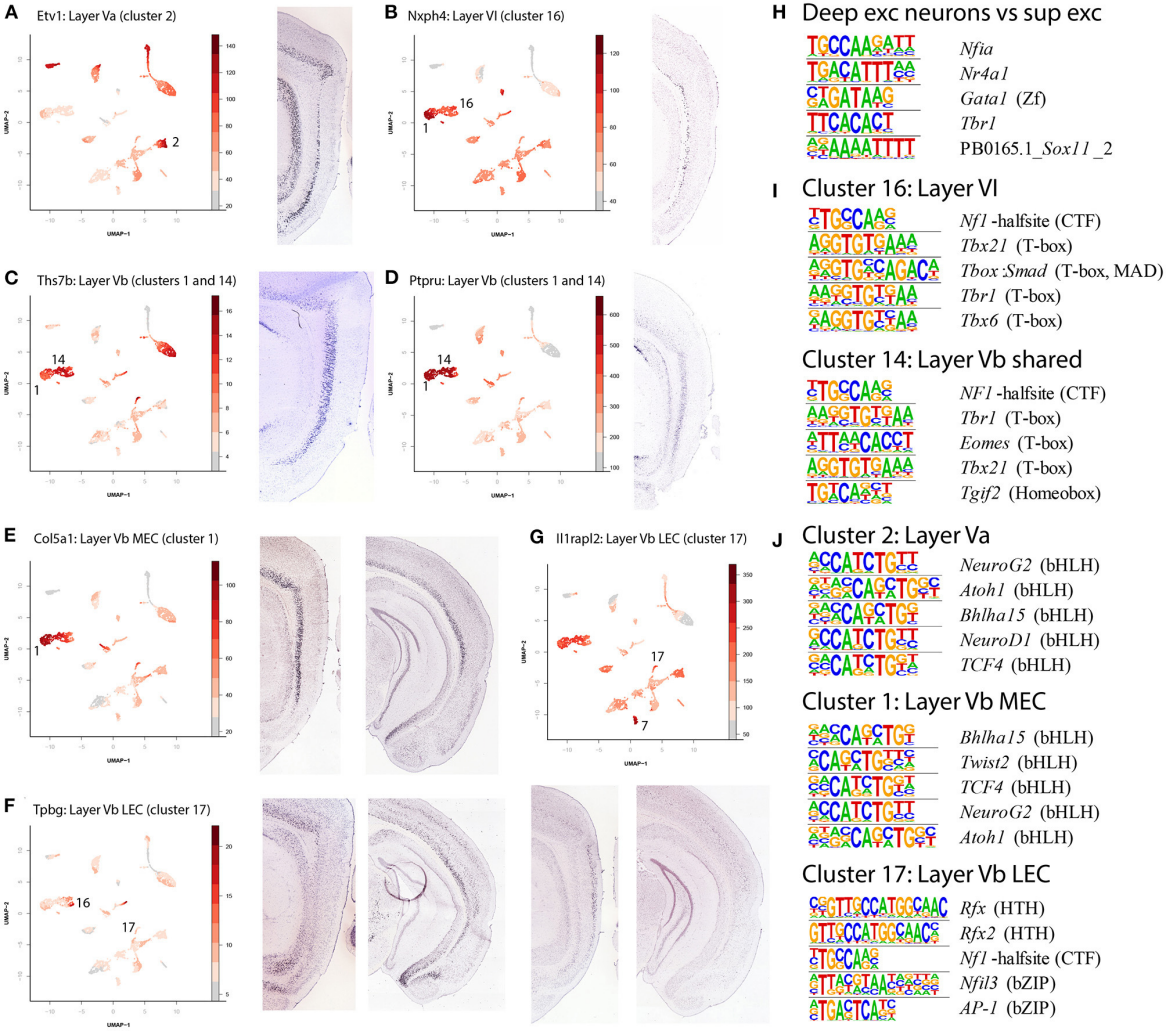
Identification of deep layer excitatory neuron clusters and enriched motifs. **(A)** Identification of Layer Va (LEC and MEC, cluster 2) cells by accessibility of *Etv1*. **(B)** Identification of Layer VI (LEC and MEC, cluster 16) cells by accessibility of *Nxph4*. **(C)** Identification of Layer Vb (LEC, MEC shared, clusters 1 and 14) cells by accessibility of *Ths7b*. **(D)** Identification of Layer Vb (LEC, MEC shared, clusters 1 and 14) cells by accessibility of *Ptpru*. **(E)** Identification of Layer Vb (MEC only, cluster 1) cells by accessibility of *Col5a1*. **(F)** Identification of Layer Vb (LEC only, cluster 17) cells by accessibility of *Tpbg*. **(G)** Identification of Layer Vb (LEC only, cluster 17) cells by accessibility of *Ilrapl2*. **(H)** Enriched motifs in the deep layers of the EC and their corresponding best matching transcription factor. Here selected DARs of all excitatory deep layer neurons were contrasted with selected DARs of all excitatory superficial layer neurons. **(I)** Enriched motifs in clusters 14 and 16. Here selected DARs of cluster 16 (LVI) were with all other non-overlapping DARs, and the same was done for cluster 14 (LVb). Note that the *Tbr1* motif is the same as the one in **(H)**, but in reverse complement. **(J)** Enriched motifs in clusters 1, 2, and 17. The contrasts are similar to the ones in **(I)**, contrasting selected DARs of one cluster with all other, non-overlapping, DARs.

Interestingly, the deep layers showed a mixture of regionally specific and regionally shared excitatory neurons. Five clusters contained excitatory cells from deep layers (clusters 1, 2, 14, 16, 17). The clusters for Layers Va (Figure 4A, cluster 2, gene *Etv1*) and VI (Figure 4B, cluster 16, gene *Nxph4*), as well as one of the LVb clusters (Figures 4C,D, cluster 14, genes *Ths7b* and *Ptpru*) contained cells from both the lateral and the medial part of EC. Conversely, clusters 1 and 17, both containing LVb cells, were either MEC or LEC specific specific (Figures 4E–G, clusters 1 and 17, genes *Col5a1, Tpbg* and *Il1rapl2*).

The combination of gene expression in the different clusters allowed us to identify each cluster with a biologically relevant cell type (Table 1). Comparing this with the hierarchical clustering (Figure 1E), we find that the shared clades are non-neurons and interneurons. This is an expected result (Tasic et al., 2018). Furthermore, we find that those clades containing superficial clusters had either LEC or MEC members. Conversely, the clusters with cells from Layers Va, Vb, and VI consisted of a mixture of LEC and MEC cells. This is in line with the observation of Ramsden et al. (2015) that LEC and MEC are more diverse in gene expression in the superficial layers compared to deep layers. Notably, we found regionally specific populations of deep excitatory neurons only in LVb.

**Table 1:** Overview of cell types in scATAC-seq clusters.

**Cluster**	**Cell class**	**Cell type**	**scRNAseq clusters**
1	Excitatory neuron	Deep: LVb (MEC)	1, 23
2	Excitatory neuron	Deep: LVa	5, 15
3	Excitatory neuron	Superficial: LII Wfs+/Calb+ (MEC)	11, 13
4	Inhibitory neuron	Vasoactive intestinal peptide expressing	10, 12, 27
5	Inhibitory neuron	Somatostatin expressing	6
6	Microglia		34
7	Excitatory neuron	Superficial: LII Reelin+ (MEC)	8
8	Excitatory neuron	Superficial: LIII (MEC)	0
9	Oligodendrocytes		31
10	Excitatory neuron	Superficial: LII Reelin+ (LEC)	3
11	Astrocytes		25
12	Putatively ependymal cells		33
13	Inhibitory neuron	Neuropeptide Y expressing	14, 24
14	Excitatory neuron	Deep: LVb	2
15	Excitatory neuron	Superficial: LII calb1+ (LEC)	9
16	Excitatory neuron	Deep: LVI	18, 19
17	Excitatory neuron	Deep: LVb (LEC)	16
18	Endothelial cells		32
19	Excitatory neuron	Superficial: LIII (LEC)	4
20	Inhibitory neuron	Parvalbumin expressing	7, 17, 26

*All clusters as identified by open chromatin profile could be identified as biologically relevant cell classes. This classification is based gene accessibility scores. The corresponding scRNAseq clusters result from numbers of scATAC cells assigned based on prediction scores. Green cells indicate excitatory neurons, red cells indicate inhibitory neurons, yellow cells indicate non-neurons*.

Using cluster specific DARs we found enrichment of different motifs in the different neuronal cell types in the deep layers, elucidating their relative relationship and possible transcription factors involved. First a comparison between the superficial and deep excitatory neuronal cell types yielded 18 motifs enriched in deep neuronal cell types (Supplementary Data Sheet 1, top 5 in Figure 4H), including *Tbr1*. Further analysis of individual cell types indicated significant overlap in the most enriched motifs between clusters 14 (LVI) and 16 (LVb shared, Figure 4I), each contrasted against DARs from all other clusters. In particular, *Tbr1* stands out. This motif is less enriched in cluster 2 (LVa), 1 and 17 (LVb specific, Figure 4J); in these clusters several basic helix-loop-helix motifs stand out (*NeuroG2, Atoh1, Tcf4*). Cluster 17 had DARs of low significance, likely resulting in a diverging picture from the other two clusters. Taken together this could modify our view on the deep layers, where a regionally shared population of LVb is more similar to LVI and a regionally specific population is more similar to LVa.

### Clustering and Identification of scRNA-Seq Cells

**Figure 5:**
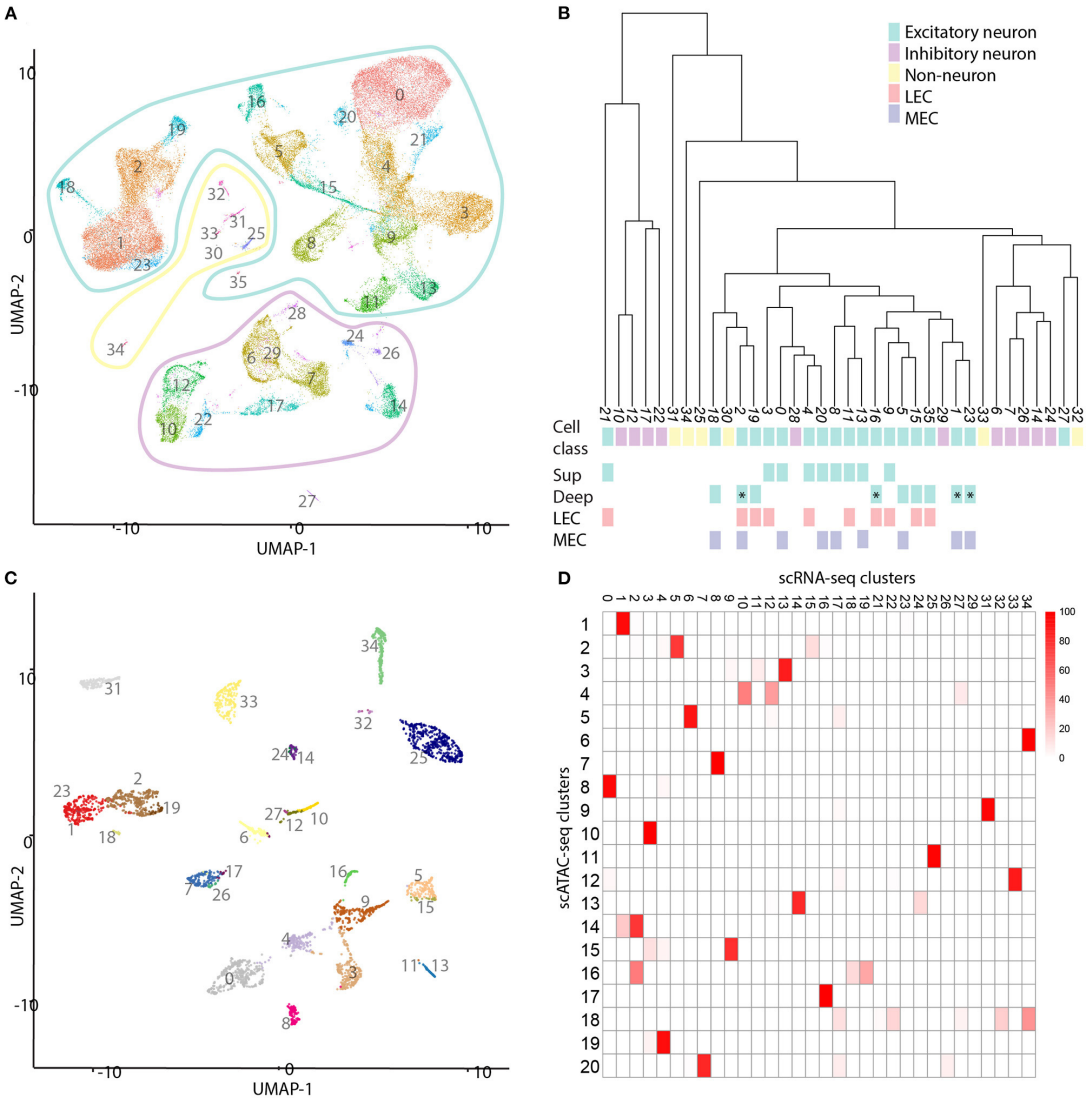
Comparison of single cell transcriptomic and chromatin data. **(A)** UMAP projection of a selection of the cells published by Yao et al. (2021). We identified 36 different clusters of cell types in this subset of data. The outlines indicate three different classes of cells: non-neurons (yellow), inhibitory neurons (magenta), and excitatory neurons (cyan). **(B)** Hierarchical dendrogram of the clusters of scRNA-seq cells. Cluster numbers, cell class, and colors correspond with those in **(A)**. LVb clusters are indicated with an asterisk. **(C)** Projection of most similar transcriptomic cell types onto the scATAC data. Each transcriptomic cell is compared to each single cell ATAC cell and the connected to the most similar one. **(D)** Heatmap of mapping scATAC-seq on scRNA-seq cells. Each cell in the heatmap indicates the percentage of scATAC cells mapping to scRNAseq clusters. The scale bar indicates percentages.

To improve our ability to identify clusters, we integrated our data with previously published transcriptomic data obtained from single cells in the entorhinal cortex (Yao et al., 2021). We used exclusively the entorhinal data obtained by the 10X pipeline, 61627 individual cells. After filtering based on percentage of mitochondrial genes (<25%) and number of features (500–8000) (Supplementary Figure 5), we continued with 59,532 individual cells (Figure 5A). We created a K nearest neighbor graph based on the top 44 principle components of the top 2,000 most variably transcribed genes (Supplementary Figure 6). This was followed by clustering using the Louvain algorithm (Blondel et al., 2008), and yielded 36 separate clusters. The majority of these clusters made biological sense based on differentially expressed genes (Supplementary Figure 7; Table 2).

**Table 2:** Transcriptomic cell types.

**scRNAseq cluster**	**#Cells**	**Cell class**	**Top DE genes**	**Cell type**	**Yao et al. clusters**	**Pcp4**	**Bcl11b**
3	4429	Neurons	Tafa1, Nptxr, Reln, Nrg1, Tafa2, Pkib	LII, Reelin (LEC)	151–161		0.53
8	2103	Neurons	Il1rapl2, Dcc, Cpa6, Gpc5, Unc13c, Fstl4	LII, Reelin (MEC)	146–150		
9	1994	Neurons	Gm32647, Rfx3, Tafa1, Nptxr, Bhlhe22, Cdh13	LII (LEC)	151–161		
11	1550	Neurons	Wfs1, Tshz2, Sgcd, Cntnap5b, Vwc2l, Khdrbs2	LII, Calbindin (LEC)	151–161		
13	1341	Neurons	Wfs1, Tshz2, Cntnap5b, Cntnap5a, Epha4, Trps1	LII, Calbindin (MEC)	146–150		
0	9016	Neurons	Igfbp5, Ntng1, 9330185C12Rik, Zfp804b, Cntnap5a, Chrm3	LIII (MEC)	135–137	1.24	
4	3819	Neurons	Fign, Trps1, Rgs4, Mgat4c, Lmo4, Pdzrn3	LIII (LEC)	135–140		
20	778	Neurons	Plch1, Cd44, Gm49906, Fermt1, Igfbp5, 9330185C12Rik	LIII (MEC)	135–137		
21	698	Neurons	Trps1, mt-Nd1, mt-Co3, mt-Atp6, Kcnip4, mt-Cytb	LIII (LEC)	135–140		
5	2721	Neurons	Etv1, Rorb, C1ql3, Gpc6, Dcn, Ncam2	LVa (MEC)	210–213		
15	1252	Neurons	Dcn, Kirrel3, 6530403H02Rik, C1ql3, Pcdh7, Cadps2	LVa (LEC)	210–213		
35	76	Neurons	Ptgfr, Npsr1, Gm2164, Teddm3, Mt2, Nts	LVa (LEC)	210–213		0.81
1	8035	Neurons	Brinp3, Thsd7b, Zfpm2, Meis2, Hs3st4, Rmst	LVb (MEC)	210–213	1.13	1.00
2	4719	Neurons	Cdh18, Vxn, Hs3st4, Pde1a, Slc1a2, Cryab	LVb (Shared MEC and LEC)	210–213	0.99	0.79
16	1233	Neurons	Nnat, Grid2, C1ql3, Tenm3, Ror1, Ntng2	LVb (LEC)	210–213	−2.12	0.29
23	572	Neurons	Zfpm2, Rmst, Rorb, Sntb1, Kcnq5, Efhd2	LVb (MEC)	210–213	0	0
18	795	Neurons	Tshz2, Cdh18, Gpc6, Pcdh17, Tox, Nxph3	LVI (MEC)	233–235	0.77	
19	787	Neurons	Frmpd4, Gm49678, C130073E24Rik, Ccn2, Kcnab1, Nxph3	LVI (LEC)	298–302	0.65	
27	330	Neurons	Dach1, Ndnf, Trp73, Nhlh2, Ebf3, Lhx1os	Excitatory	Unknown	0.72	
17	904	Neurons	Nxph1, Kcnc2, Galntl6, Grik1, Erbb4, Cntnap2	PV+ interneurons	120, 121, 123		
7	2187	Neurons	Kcnc2, Erbb4, Nxph1, Slc6a1, Calb1, Btbd11	PV+ interneurons	120, 121, 123		
26	342	Neurons	Pthlh, Mxra7, Cox6a2, Dlx1as, C1ql1, Unc5b	PV+ interneurons	120, 121, 123		
22	663	Neurons	Adarb2, Erbb4, Galntl6, Cntnap2, Sox2ot, Calm2	VIP+ interneurons	42–62		
28	302	Neurons	Gad2, Slc32a1, Kcnmb2, Gad1, Slc6a1, Sox2ot	VIP+ interneurons	42–62		
10	1563	Neurons	Vip, Adarb2, Cxcl14, Synpr, Erbb4, Igf1	VIP+ interneurons	42–62		
12	1433	Neurons	Cnr1, Adarb2, Cxcl14, Npas3, Cadps2, Col25a1	VIP+ interneurons	42–62		
24	387	Neurons	Hapln1, Slc6a1, Id2, Gad1, Gad2, Zfp536	NPY+ interneurons	10, 27, 28, 32, 39		0.67
14	1297	Neurons	Fgf13, Gad2, Gad1, Kit, Npy, Reln	NPY+ interneurons	10, 27, 28, 32, 39		0.68
29	290	Neurons	Arx, Kcnmb2, Nxph1, Crhbp, Slc32a1, Sox6	SST+ interneurons	87, 105, 106, 107		0.32
6	2656	Neurons	Sst, Nxph1, Npy, Grin3a, Grik1, Gad2	SST+ interneurons	87, 105, 106, 107		
25	384	Non-neurons	Slc1a3, Gpc5, Atp1a2, Mt2, Luzp2, Plpp3	Astrocytes	376–378		
30	242	Non-neurons	Tcf4, Zfp365, Ralbp1, Rtf1, Rab11fip2, Nucks1	Non-neurons	Unknown		
31	229	Non-neurons	Plp1, Mobp, Cnp, Cldn11, Gatm, Ptgds	Oligodendrocytes	365–375		
32	168	Non-neurons	Flt1, Ebf1, Rgs5, Slco1a4, Cldn5, Adgrl4	Endothelial	379		
33	129	Non-neurons	Pdgfra, Olig1, Gpr17, S100a16, Sox10, C1ql1	Ependymal	Unknown		
34	108	Non-neurons	C1qa, Cx3cr1, C1qb, C1qc, Tyrobp, Selenop	Microglia	386–388		

*Most clusters, as identified by transcriptomic profile, could be linked to biologically relevant cell classes. Likely correspondence between cell types identified by Yao et al. with those found here, was done manually based on most represent the relative enrichment (log Fold Change, labeled green for positive and purple for negative or zero) of these genes in corresponding scRNAseq clusters compared to all other clusters. In the central columns, green cells indicate excitatory neurons, red cells indicate inhibitory neurons, yellow cells indicate non-neurons*.

Using the differentially expressed genes in combination with previously described marker genes, we were able to link transcriptomic clusters to biologically relevant cell types (Figure 5A; Supplementary Figure 7; Supplementary Table 5; Table 2). Of the 36 clusters, 30 could be identified as neuronal cell types. After hierarchical clustering on averaged gene expression (per cluster), several small non-neuronal clusters (30, 32, 33) were classified in unexpected clades. This is likely not due to mitochondrial genes (Figure 5; Supplementary Figure 8). Of the neuronal cell types, 11 have transcriptional profiles of different types of inhibitory neurons, while the rest have profiles of excitatory neurons. The excitatory cell types can be separated into superficial (nine clusters) and deep layer (nine clusters) cell types (Figure 5B). The deep cell types comprise LVa (three clusters), LVb (four clusters) and LVI (two clusters). Even though this is fewer than Yao et al. find based on the same (but expanded with additional cortical regions) data, the clusters are biologically relevant.

### Integration of scATAC-Seq Cells and scRNA-Seq Cells

Next, we compared the transcriptional cell types with those based on chromatin (Figures 5C,D; Supplementary Data Sheet 3). To do this, on each cell from the scATAC-seq data, we mapped the most similar scRNA-seq cell type. We found that primarily the inhibitory cell types as identified by chromatin are more tessellated based on transcriptomic data. In particular the *VIP* and *PV*-expressing cell types, which each subcluster into three different groups. Additionally, several of the excitatory scATAC-seq clusters are subclustered based on transcriptomic data. Notably both the LVa and LVI chromatin clusters subdivide into two transcriptomic clusters each. Here LVa comprises transcriptomic clusters 5 and 15, corresponding largely to MEC and LEC cells respectively. The third transcriptional cluster (35) did not map to any chromatin clusters, but gene expression shows this cluster is LVa specific. LVI comprises transcriptomic clusters 18 and 19 corresponding largely to MEC and LEC cells respectively. Meaning, these subdivisions are likely biologically relevant and correspond to the different subdivisions of the EC. Interestingly, the LVb cluster shared between LEC and MEC is not subdivided further based on transcriptomic data. Even though the MEC specific LVb chromatin cluster is separated into two components based on transcriptomic data. This solidifies the existence of a shared excitatory cell type between LEC and MEC in LVb, in addition to the region specific LVb cell types.

In addition to identifying the top DE genes as contrasts between single clusters and all other clusters (Supplementary Table 5; Table 2), using transcriptomic data we were able to find DE genes between particular clusters (Supplementary Tables 2–4; Supplementary Data Sheet 2). When applied to the LVb clusters, the DE genes were largely overlapping with those found for clusters contrasted with all other clusters (Supplementary Tables 2, 3; Table 2). Meaning that the shared LVb cluster is marked by high expression of *Vxn, Cdh18*, and *Pde1a*. The contrast with the unique LVb clusters adds genes to this list, including *Prex2, Ogfrl1*, and *Plcl1*. The MEC specific LVb clusters are marked by *Brinp3, Thsd7b, Zfpm2, Rmst*, and *Rorb*, while the LEC specific LVb cluster is marked by *Nnat, Grid2, C1ql3*, and *Tenm3*. The contrast with the shared LVb clusters reveals the additional genes *Khdrbs2, Cacnb4*, and *Rprm*. Interestingly, similar to the corresponding motif, transcription factor *Tbr1* is enriched in transcriptional clusters 1, 2, 8, 18, 19, 23, and 29 (Supplementary Table 5), linked to amongst others LVI, LVb (MEC and shared), LII (*Reelin*+, MEC) excitatory neurons but not LVb (LEC) and LVa neurons. When investigating the numbers of DE genes within layers, contrasting the medial and lateral parts of the EC, no clear difference becomes apparent between superficial and deep cell types (Figure 5; Supplementary Data Sheet 2, Supplementary Table 4). These contrasts can be used however, to identify additional genes identifying regionally specific cell types (Supplementary Table 4). This combined epigenomic and transcriptomic data may lead to strategies to genetically target these particular cell types.

Further investigation of DE genes can give insight to the associations between cell types identified here and those described previously based on particular markers. *Purkinje Cell Protein 4* (*Pcp4*) is enriched specifically in LIII MEC and LVb EC cells (Lein et al., 2007; Tang et al., 2015; Ohara et al., 2018). In our data, this gene is enriched in clusters corresponding to LIII MEC cells (cluster 0), MEC specific LVb cells (cluster 1), shared LVb cells (cluster 2), and LVI cells (LEC and MEC specific, clusters 18 and 19, Table 2). Interestingly, *Pcp4* is not enriched in two clusters of regionally specific LVb cells, with the LEC cluster (16) showing a negative LogFold enrichment (-2.12) and the MEC cluster (23) showing no significant deviation (Table 2; Supplementary Table 5). Another previously described marker is *COUP-TF Interacting Protein 2* [*Ctip2* (Surmeli et al., 2015), also known as *Bcl11b*], which was enriched in clusters 1, 2, 3, 14, 16, 24, 29, and 35. Amongst others, these clusters correspond to MEC and LEC specific LVb and shared LVb cells, and various interneurons. Combining these two previously known markers, we see that the populations of LEC specific LVb cells (cluster 16) is enriched only with *Ctip2* and a population of MEC specific LVb cells with neither (cluster 23, Table 2). The shared LVb and MEC specific LVb populations (clusters 1 and 2) are enriched for both genes. These previously well-described marker genes in combination with the data presented here may be used in the future to tease out the functional differences of the different cell types.

## Discussion

Here we present new data to classify entorhinal cortex cell types. We find that largely the cell types correspond to previously described classifications, but that LVb has populations specific to LEC, specific to MEC and a third one shared between the two subdivisions. When combined with previously published transcriptomic data, we find that a shared population is unique to LVb. With their intra-entorhinal projections toward LVa, LII, and LIII, these neurons fall into two distinct connectivity and functional types (Ohara et al., 2018). The first type mediates a hippocampal output circuit with LVa projecting onwards to telencephalic extra-hippocampal areas while the second type mediates a feedback projecting toward layers II and III (Dolorfo and Amaral, 1998; Surmeli et al., 2015).

In our scATAC-seq data we found clusters for all major cell types, but the deep layer clusters are more shared between LEC and MEC than the superficial layer ones. In the superficial layers, both LEC and MEC had three separate clusters. Contrary to this, layers LVa, LVb, and LVI have shared (LEC+MEC cells) clusters. This resonates with the finding by Ramsden et al. (2015) that these two regions are more similar in their deep layers compared to the superficial layers. Investigation of motifs in DARs shows that the shared LVb population has more similarity to LVI while the regionally specific ones have more similarity to LVa. The transcription factor *Tbr1* may play a role in this differentiation. The LEC and MEC are different in their functional cell types as well as their intrinsic and extrinsic connectivity. Current data suggests the differences in functionality and intrinsic connectivity are present primarily in in the superficial layers (Hafting et al., 2005; Couey et al., 2013; Leitner et al., 2016; Tsao et al., 2018). This corresponds to the larger variability of chromatin cell types in the superficial layers between the two regions.

Transcriptomic data finds a higher resolution of cell types than our scATAC-seq data. There may be either technical or biological reasons for this. The two datasets are not equivalent, with the most obvious difference the sheer number of cells in the transcriptomic data being more than an order of magnitude larger. This can obviously lead to the detection of more clusters, and an upscaled scATAC-seq experiment could lead to the detection of more cell types based on chromatin accessibility. Moreover, there are several possible technical differences between the two techniques, for example the more quantitative nature of scRNA-seq and the faster deterioration of chromatin compared to mRNA. Besides the technical limitations, there may also be biological reasons for the difference. It is possible that transcriptionally different cell types share a more similar chromatin profile. The technique used here to investigate chromatin profile, scATAC-seq, is rather one-dimensional, where the open or closed state of the chromatin may have several meanings and has several more dimensions (Jiang and Mortazavi, 2018). Addition of more chromatin marks may fill in these additional dimensions (Ernst et al., 2011).

Both transcriptomic data and scATAC-seq data indicate LVb has a shared population in addition to LEC and MEC specific ones. The LVb cells receive projections from the hippocampus. From there they project to LVa, LII, and LIII, where they facilitate re-entry to the hippocampus or transmission to other cortical regions (Witter et al., 1989; Iijima et al., 1996). This circuit is thought to play a role in consolidation of transient information (Buzsaki, 1996; Eichenbaum et al., 2012). In this scheme, transient information is held by the entorhinal-hippocampal network, while the consolidated information is stored in the neocortex. Notably, the dorsal LEC LVb has a much stronger projection toward LVa than the dorsal MEC (Ohara et al., 2021). Conversely, other intrinsic circuits (LVb to LII and LIII) are very similar between the two entorhinal regions (Ohara et al., 2018). Therefore, it seems reasonable to postulate that the shared LVb population in our scATAC-seq data corresponds to these superficial layer projecting cells. Whereas, the region-specific populations correspond to the LVa projecting LVb cells in LEC and to another LVb population in MEC. As noted by Ohara et al., these different populations are likely to have different functional roles in systems consolidation. Future tools targeting either one of the three populations may give an integrated view on their identity, anatomy, connectivity, and functionality.

## Data Availability Statement

The original contributions presented in the study are publicly available. This data can be found here: https://www.ncbi.nlm.nih.gov/bioproject/?term=PRJNA786429. The SRA accessions can be found at: https://www.ncbi.nlm.nih.gov/sra?linkname=bioproject_sra_all&from_uid=786429.

## Ethics Statement

The animal study was reviewed and approved by Norwegian Animal Welfare Act and the European Convention for the Protection of Vertebrate Animals used for Experimental and Other Scientific Purposes.

## Author Contributions

SB and CK conceived and planned this study. SB and LO analyzed data. SB, LO, and CK wrote the manuscript. All authors contributed to the article and approved the submitted version.

## Funding

This work was supported by the FRIPRO ToppForsk grant Enhanced Transgenics (90096000) of the Research Council of Norway, the Kavli Foundation, the Centre of Excellence scheme of the Research Council of Norway—Centre for Biology of Memory and Centre for Neural Computation, The Egil and Pauline Braathen and Fred Kavli Centre for Cortical Microcircuits, and the National Infrastructure scheme of the Research Council of Norway—NORBRAIN. The Genomics Core Facility was funded by the Faculty of Medicine and Health Sciences at NTNU and Central Norway Regional Health Authority.

## Conflict of Interest

The authors declare that the research was conducted in the absence of any commercial or financial relationships that could be construed as a potential conflict of interest.

## Publisher's Note

All claims expressed in this article are solely those of the authors and do not necessarily represent those of their affiliated organizations, or those of the publisher, the editors and the reviewers. Any product that may be evaluated in this article, or claim that may be made by its manufacturer, is not guaranteed or endorsed by the publisher.

## Supplementary Material

Supplementary Figure 1 Selection of legitimate nuclei. **(A–D)** Projection of all cells before filtering. Cells within the bounding boxes (promoter ratio 0.05–0.30, UMI 4–5.5) were selected for further analysis.

Supplementary Figure 2 Clustering metrics and quality control metrics. **(A)** Projection of single cells on eigen vector axis. Each panel uses two eigen vectors. The top 20 eigen vectors were selected as input for the KNN graph and community detection. **(B)** Clusters and cluster numbers for reference. **(C)** Cells labeled with the fraction of reads in peaks. **(D)** Cells labeled with the fraction of duplicate reads. **(E)** Cells labeled with the read depth. **(F)** Numbers of cells for each cluster and each sample.

Supplementary Figure 3 Identification of cell classes. **(A)** Identification of different cell classes and types by the accessibility of particular genes. In the case of **(A)** the gene is C1qc, which shows a high gene accessibility score in cluster 6, identifying this cluster as microglia. This is repeated for all major cell classes. **(B)** Hapln2; oligodendrocytes. **(C)** Flt1; endothelial cells. **(D)** Lcat; astrocytes. **(E)** Ccdc153; potentially ependymal cells. **(F)** Dynlrb2; potentially ependymal cells. **(G)** Snap25; neuronal cells. **(H)** Pnoc; inhibitory neurons. **(I)** Slc17a7a; excitatory neurons. **(J)** Motifs found in inhibitory and excitatory cell types. Here contrasts were set between DARs for all inhibitory cells (clusters 4, 5, 13, 20) and all excitatory cells (clusters 1, 2, 3, 7, 8, 10, 14, 15, 16, 17, 19).

Supplementary Figure 4 Relative accessibility of DARs. The scale bar represents the mean Z-score of all DARs per cluster.

Supplementary Figure 5 Clustering and quality control metrics for the transcriptomic data.

Supplementary Figure 6 Top variable genes in the 50 principle components. The green box indicates PC44.

Supplementary Figure 7 Top six variable genes in each cluster.

Supplementary Figure 8 Percentage of mitochondrial genes per cluster and per cell.
