# Supplementary figures and images for: Single Cell Transcriptomic and Chromatin Profiles Suggest Layer Vb Is the Only Layer With Shared Excitatory Cell Types in the Medial and Lateral Entorhinal Cortex
Source: Front Neural Circuits. 2022 Jan 26;15:806154. doi: 10.3389/fncir.2021.806154 (PMC8826650; doi:10.3389/fncir.2021.806154)

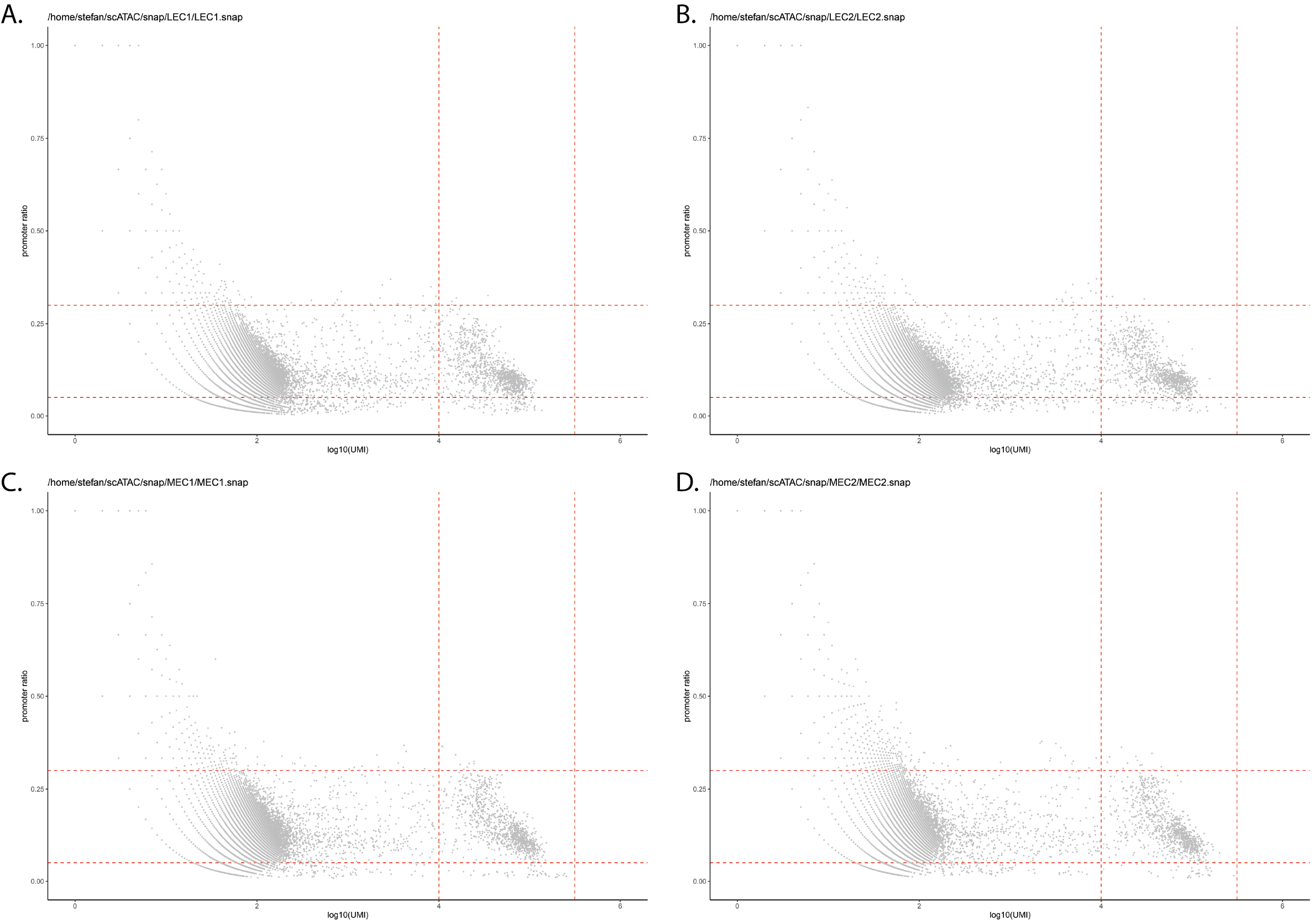

Supplement: Supplementary Figure 1 — Selection of legitimate nuclei. (A–D) Projection of all cells before filtering. Cells within the bounding boxes (promoter ratio 0.05–0.30, UMI 4–5.5) were selected for further analysis. [file Image_1.TIF]

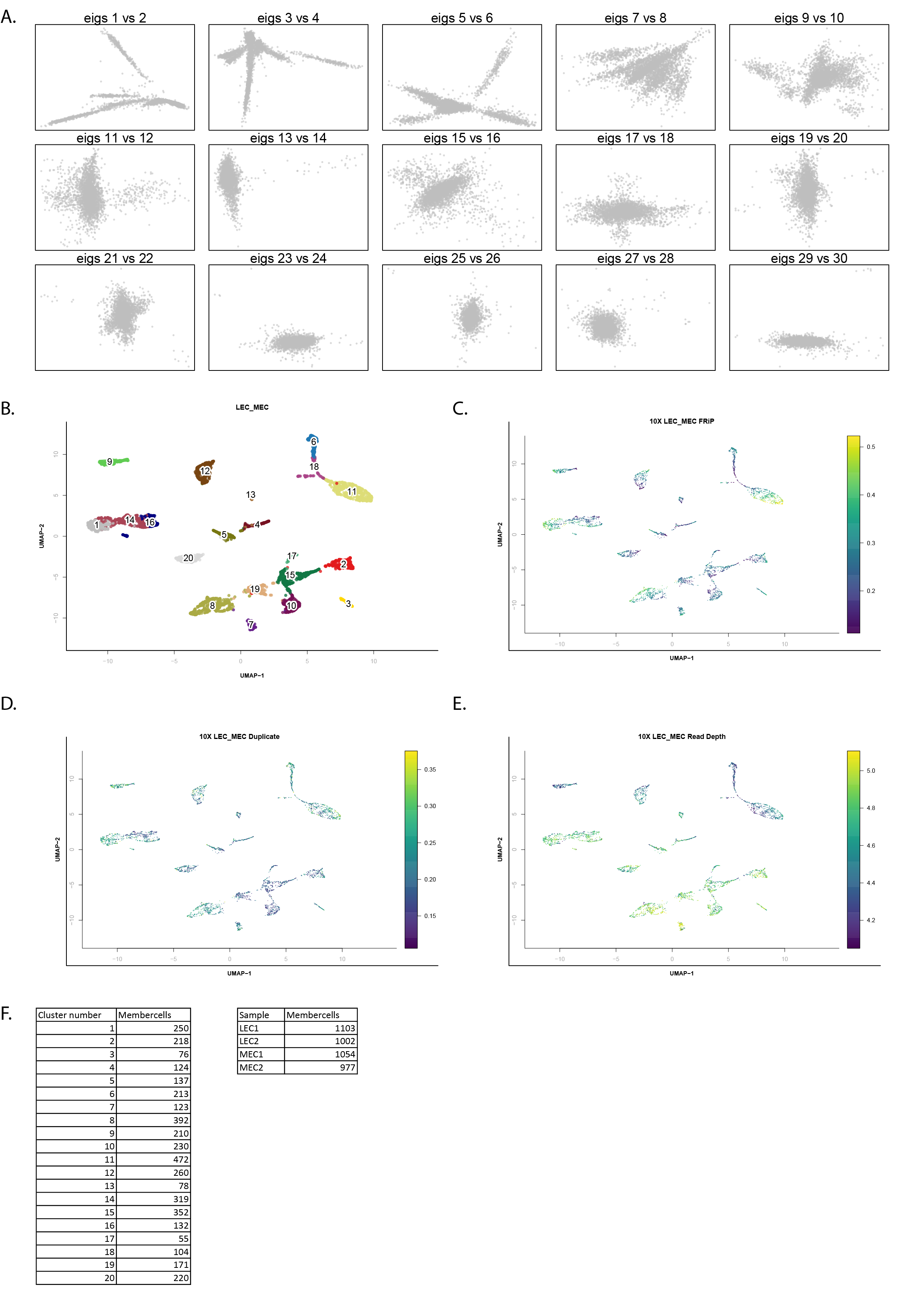

Supplement: Supplementary Figure 2 — Clustering metrics and quality control metrics. (A) Projection of single cells on eigen vector axis. Each panel uses two eigen vectors. The top 20 eigen vectors were selected as input for the KNN graph and community detection. (B) Clusters and cluster numbers for reference. (C) Cells labeled with the fraction of reads in peaks. (D) Cells labeled with the fraction of duplicate reads. (E) Cells labeled with the read depth. (F) Numbers of cells for each cluster and each sample. [file Image_2.TIF]

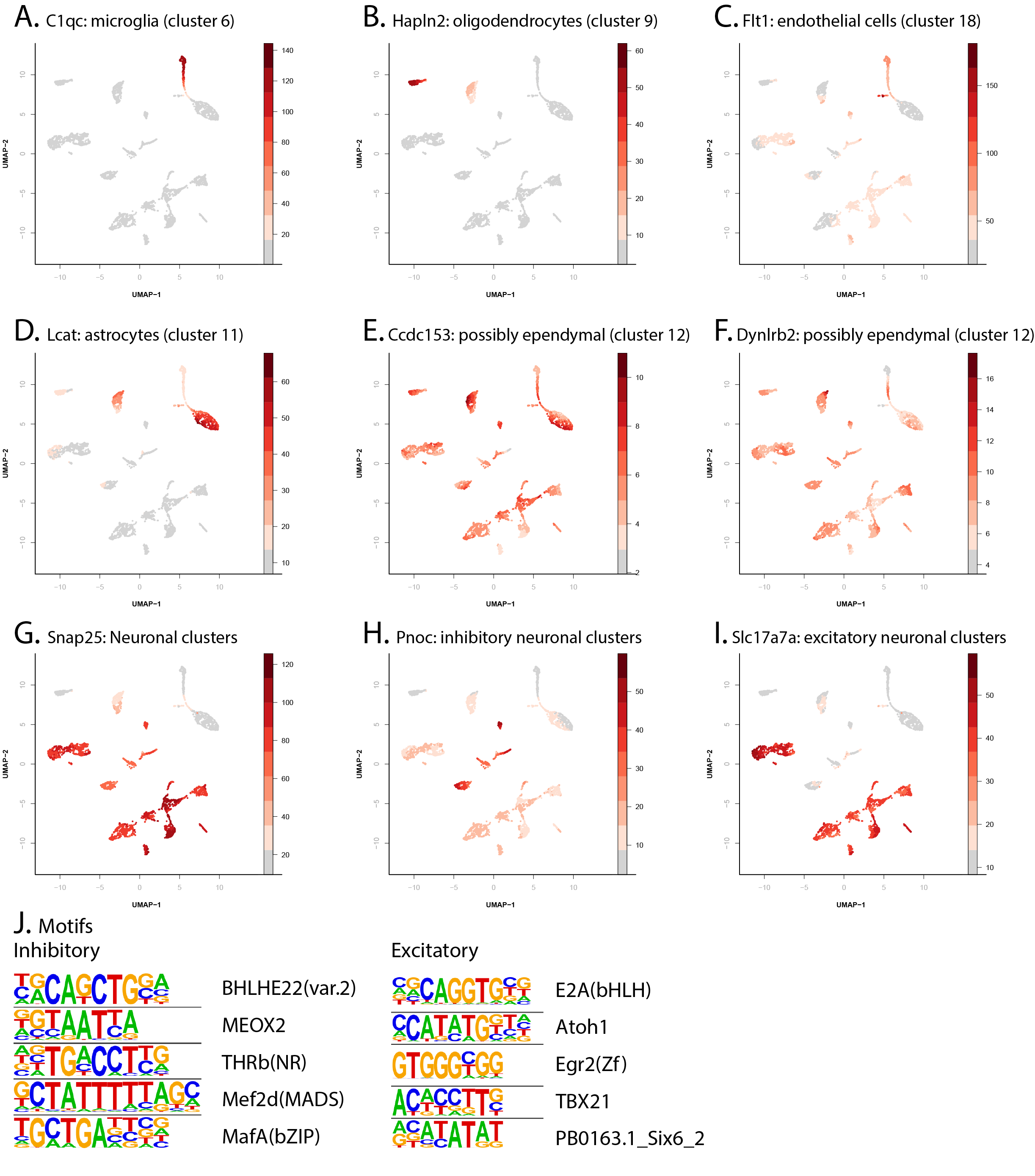

Supplement: Supplementary Figure 3 — Identification of cell classes. (A) Identification of different cell classes and types by the accessibility of particular genes. In the case of (A) the gene is C1qc, which shows a high gene accessibility score in cluster 6, identifying this cluster as microglia. This is repeated for all major cell classes. (B) Hapln2; oligodendrocytes. (C) Flt1; endothelial cells. (D) Lcat; astrocytes. (E) Ccdc153; potentially ependymal cells. (F) Dynlrb2; potentially ependymal cells. (G) Snap25; neuronal cells. (H) Pnoc; inhibitory neurons. (I) Slc17a7a; excitatory neurons. (J) Motifs found in inhibitory and excitatory cell types. Here contrasts were set between DARs for all inhibitory cells (clusters 4, 5, 13, 20) and all excitatory cells (clusters 1, 2, 3, 7, 8, 10, 14, 15, 16, 17, 19). [file Image_3.TIF]

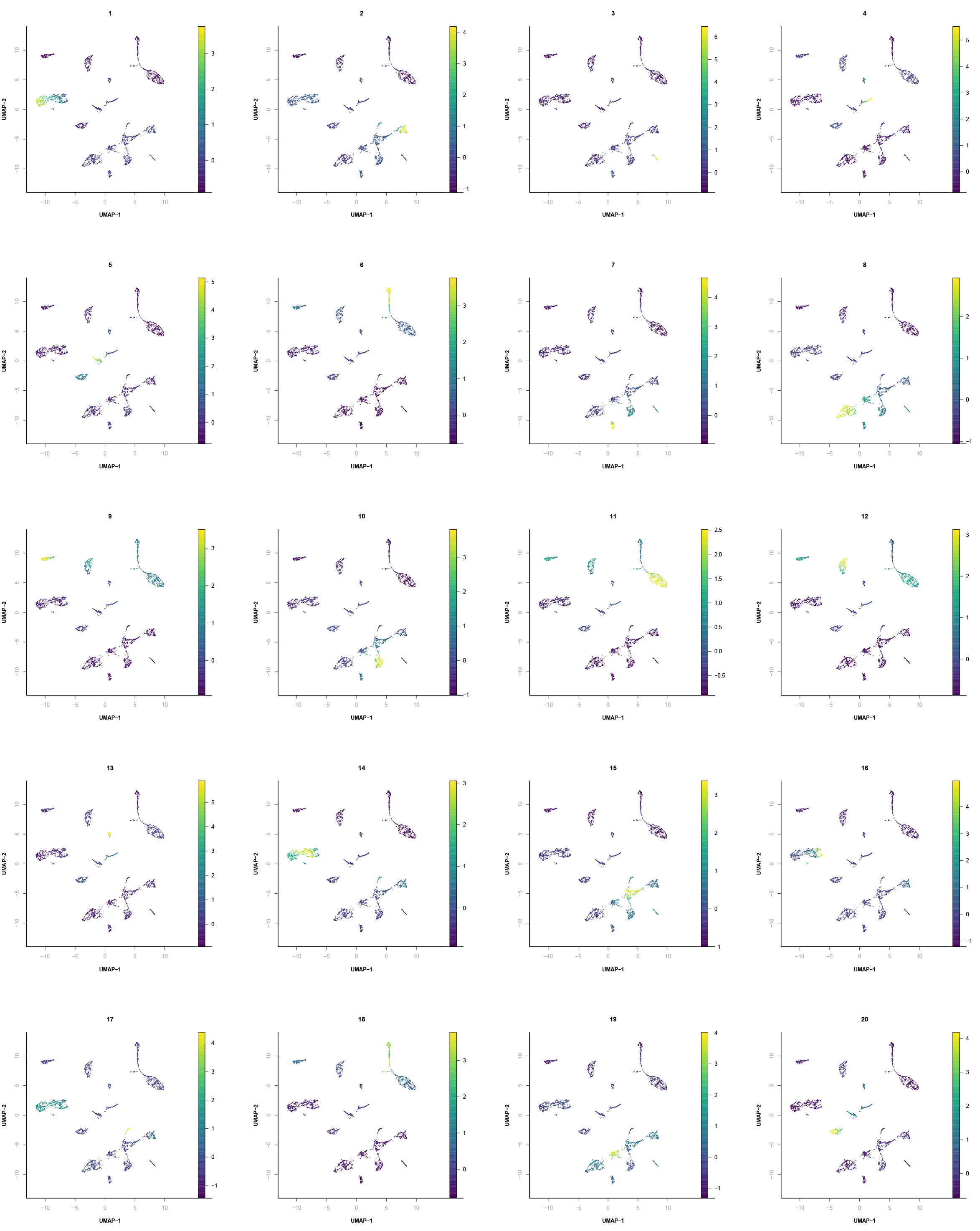

Supplement: Supplementary Figure 4 — Relative accessibility of DARs. The scale bar represents the mean Z-score of all DARs per cluster. [file Image_4.TIF]

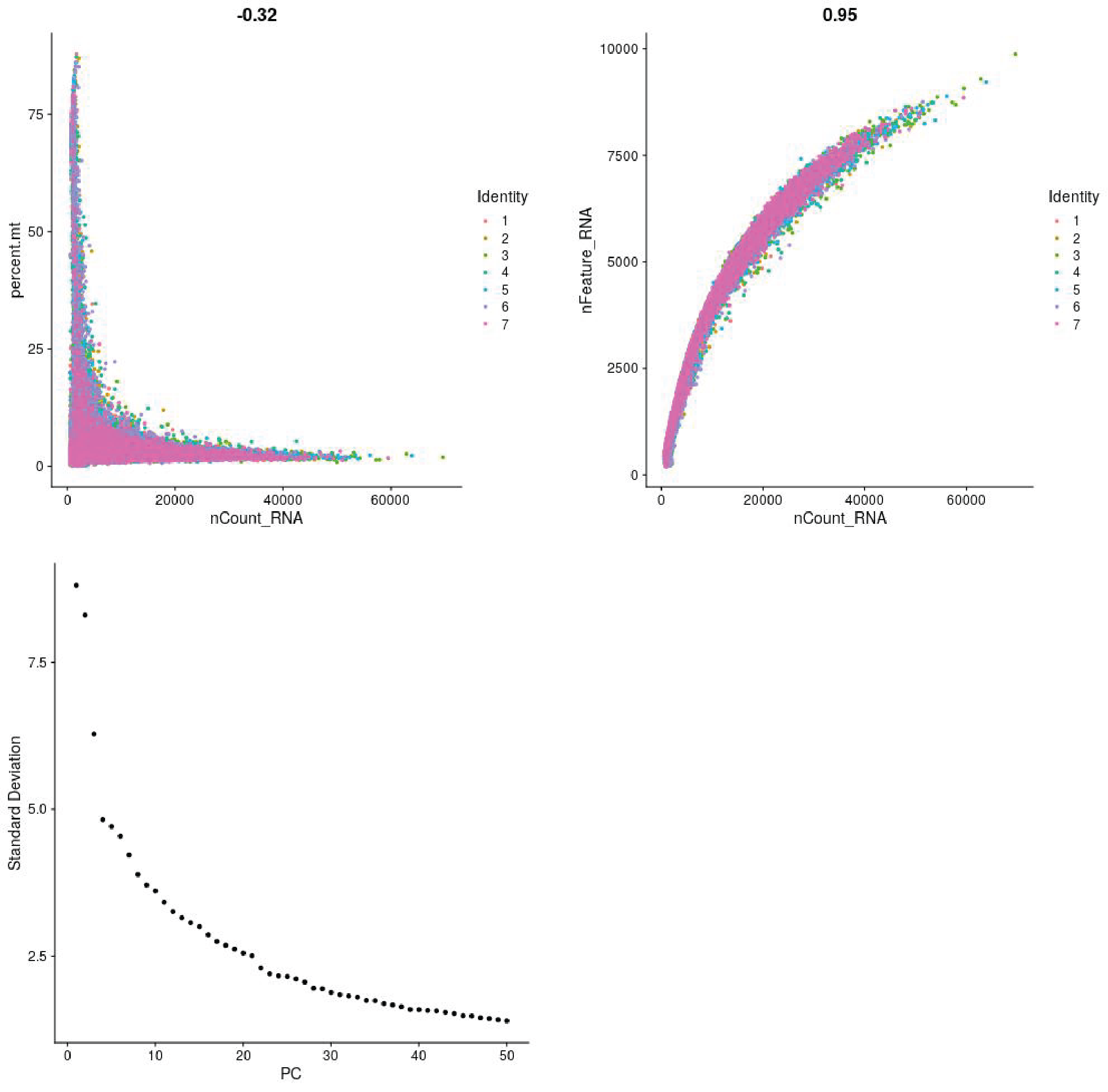

Supplement: Supplementary Figure 5 — Clustering and quality control metrics for the transcriptomic data. [file Image_5.tif]

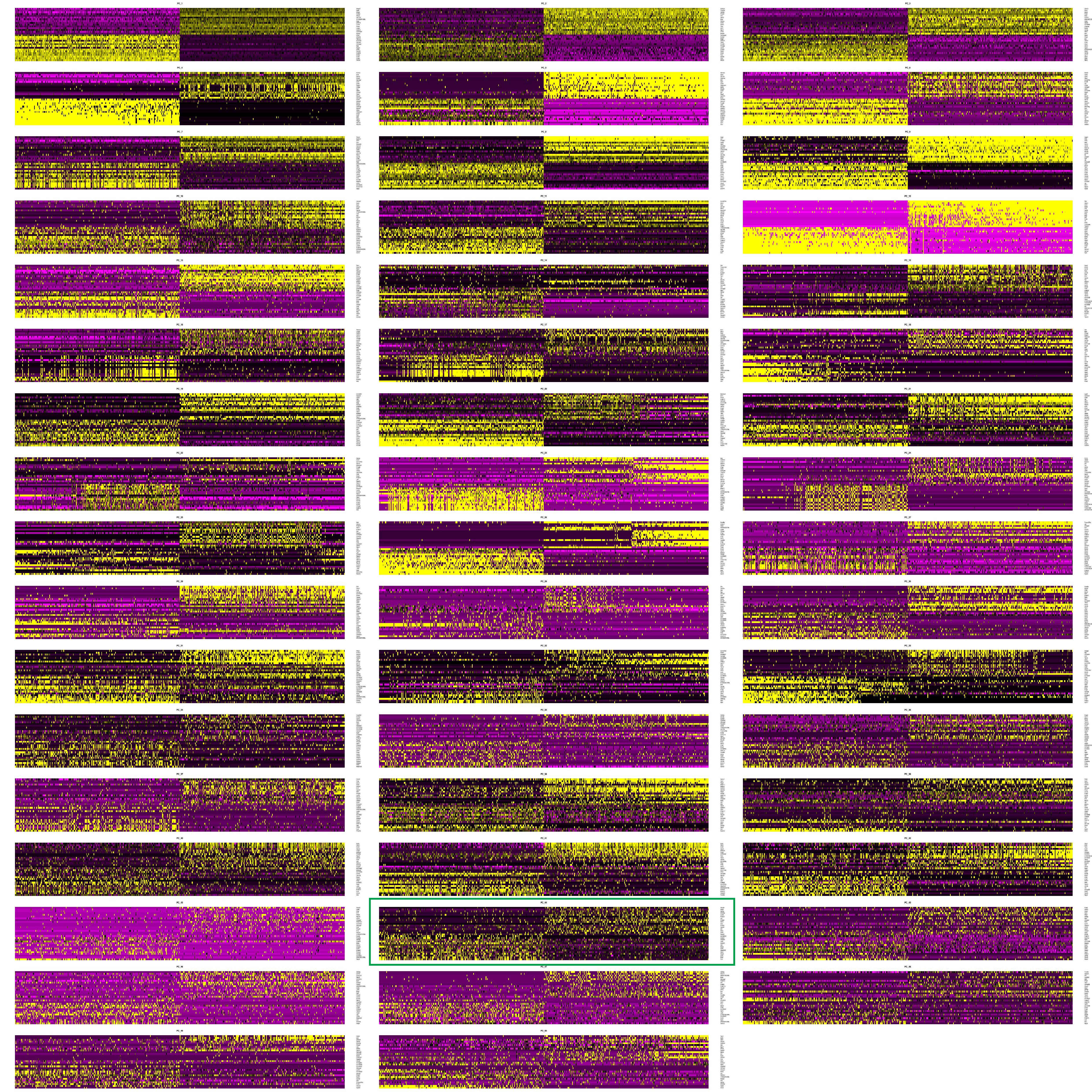

Supplement: Supplementary Figure 6 — Top variable genes in the 50 principle components. The green box indicates PC44. [file Image_6.jpg]

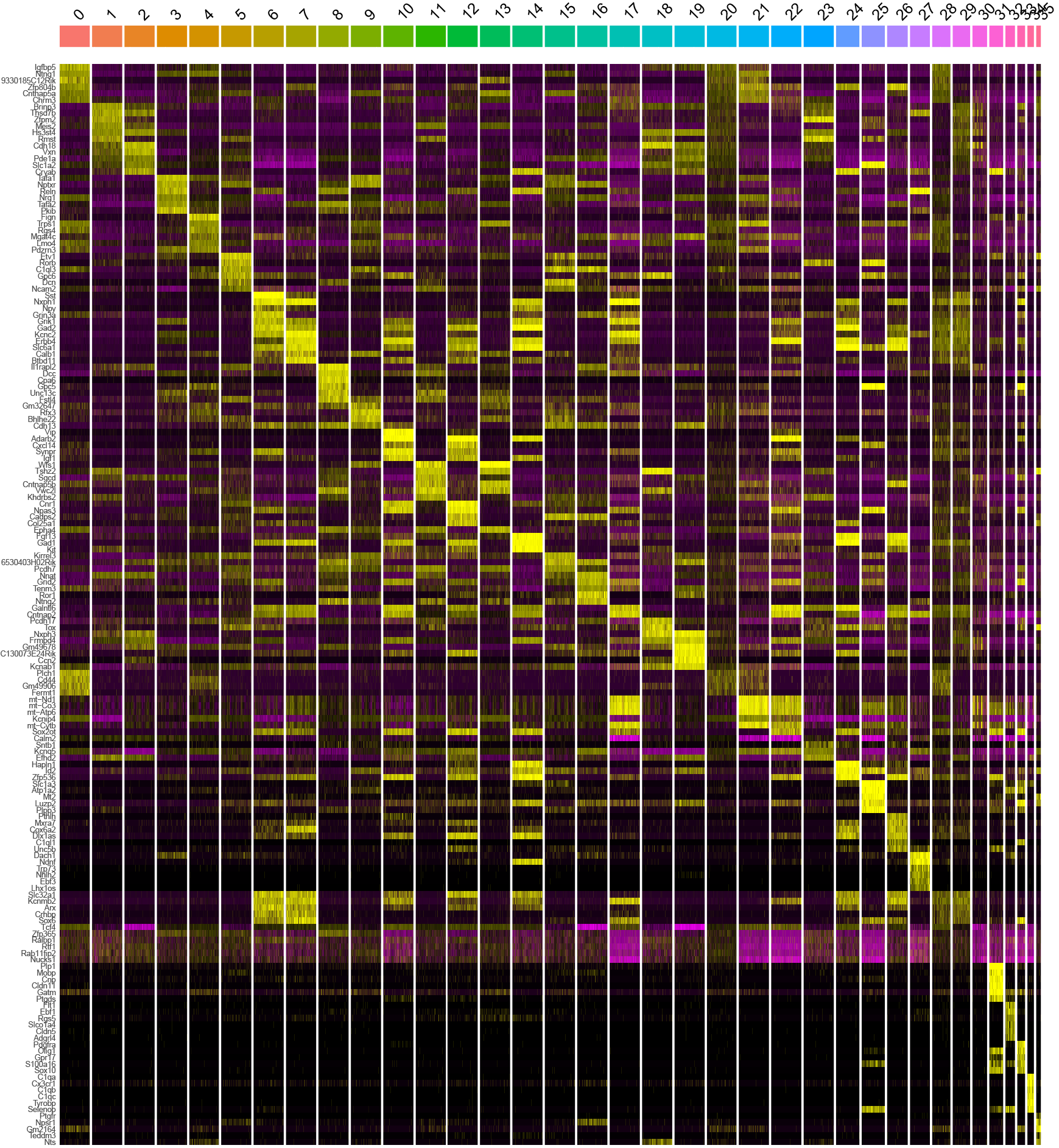

Supplement: Supplementary Figure 7 — Top six variable genes in each cluster. [file Image_7.TIF]

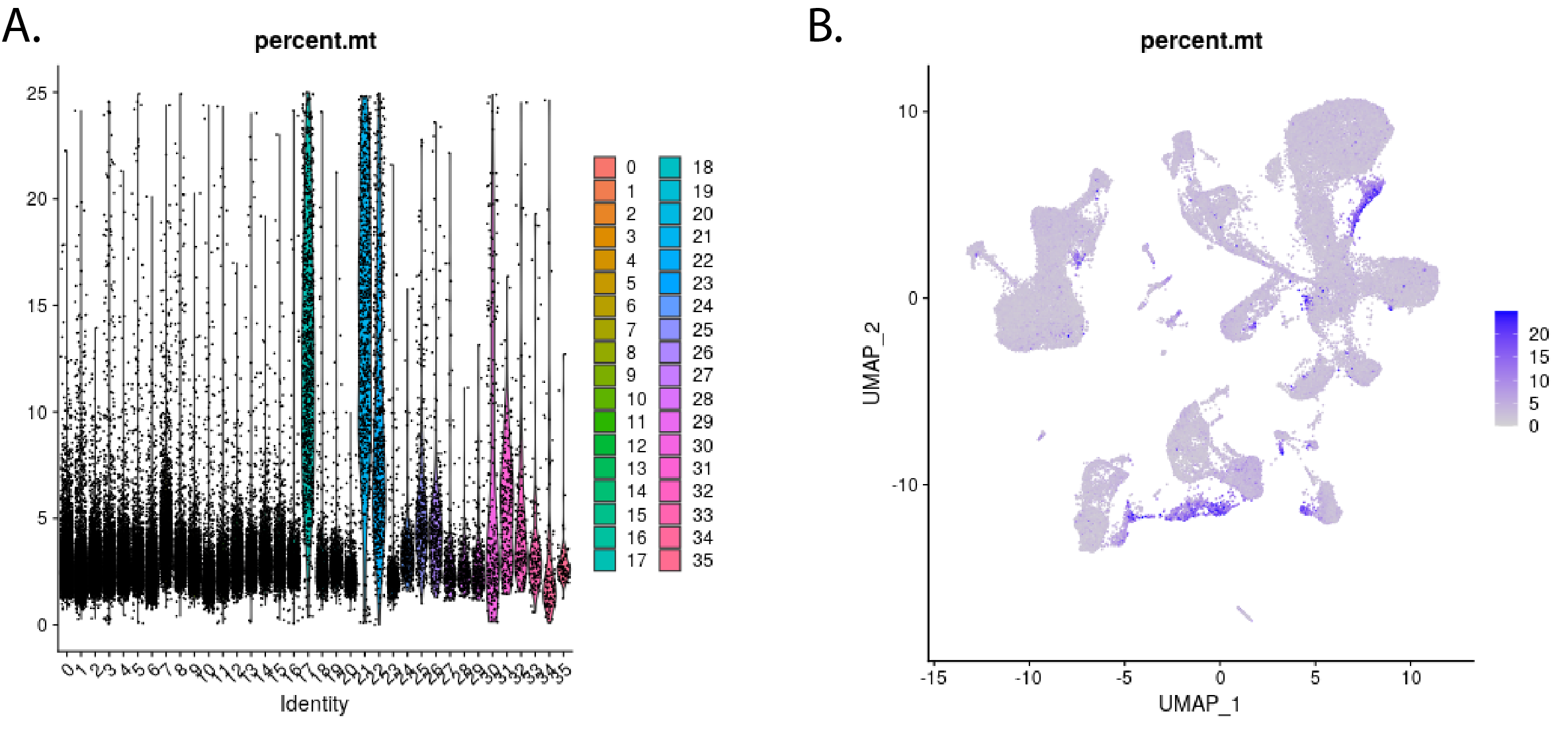

Supplement: Supplementary Figure 8 — Percentage of mitochondrial genes per cluster and per cell. [file Image_8.TIF]
