# Supplementary material for: Single Cell Transcriptomic and Chromatin Profiles Suggest Layer Vb Is the Only Layer With Shared Excitatory Cell Types in the Medial and Lateral Entorhinal Cortex
Source: Front Neural Circuits. 2022 Jan 26;15:806154. doi: 10.3389/fncir.2021.806154 (PMC8826650; doi:10.3389/fncir.2021.806154)
Supplement: Supplementary file 14 [file Data_Sheet_1.ZIP › 10/knownResults.html]

10 - Homer Known Motif Enrichment Results


# Homer Known Motif Enrichment Results (10)

Homer *de novo* Motif Results  
Gene Ontology Enrichment Results  
Known Motif Enrichment Results (txt file)  
Total Target Sequences = 7038, Total Background Sequences = 42099

|  |  |  |  |  |  |  |  |  |  |  |  |
| --- | --- | --- | --- | --- | --- | --- | --- | --- | --- | --- | --- |
| Rank | Motif | Name | P-value | log P-pvalue | q-value (Benjamini) | # Target Sequences with Motif | % of Targets Sequences with Motif | # Background Sequences with Motif | % of Background Sequences with Motif | Motif File | SVG |
| 1 | T C A G A C G T T C G A T A G C A G T C C G T A A C T G G T A C A C G T A C T G A T C G A G T C | Atoh1(bHLH)/Cerebellum-Atoh1-ChIP-Seq(GSE22111)/Homer | 1e-596 | -1.374e+03 | 0.0000 | 2614.0 | 37.14% | 5300.3 | 12.59% | motif file (matrix) | svg |
| 2 | C T A G T C G A T G A C A G T C C G T A A C T G G T A C A C G T A C T G A C T G | BHLHA15(bHLH)/NIH3T3-BHLHB8.HA-ChIP-Seq(GSE119782)/Homer | 1e-571 | -1.316e+03 | 0.0000 | 3152.0 | 44.79% | 7662.7 | 18.19% | motif file (matrix) | svg |
| 3 | T C G A T G A C G T A C C G T A C A G T T G A C A C G T A C T G A G C T A G C T | NeuroG2(bHLH)/Fibroblast-NeuroG2-ChIP-Seq(GSE75910)/Homer | 1e-543 | -1.251e+03 | 0.0000 | 3357.0 | 47.70% | 8804.7 | 20.91% | motif file (matrix) | svg |
| 4 | T C G A A G T C C G T A A T C G T A G C A C G T A C T G A G C T A C G T A G T C | Ptf1a(bHLH)/Panc1-Ptf1a-ChIP-Seq(GSE47459)/Homer | 1e-521 | -1.201e+03 | 0.0000 | 4232.0 | 60.13% | 13363.0 | 31.73% | motif file (matrix) | svg |
| 5 | C T A G A G T C T A C G T A C G T G A C C G T A A C T G T A G C G C A T C A T G A T G C A G C T | Ascl1(bHLH)/NeuralTubes-Ascl1-ChIP-Seq(GSE55840)/Homer | 1e-515 | -1.186e+03 | 0.0000 | 2863.0 | 40.68% | 6862.1 | 16.29% | motif file (matrix) | svg |
| 6 | T G A C A G T C C G T A A C T G G T A C A C G T A C T G A C G T G A C T G A T C | Twist2(bHLH)/Myoblast-Twist2.Ty1-ChIP-Seq(GSE127998)/Homer | 1e-511 | -1.178e+03 | 0.0000 | 3562.0 | 50.61% | 10051.2 | 23.87% | motif file (matrix) | svg |
| 7 | T C A G T G A C G T A C C G T A A C G T T G A C A C G T T C A G A G C T G A C T | NeuroD1(bHLH)/Islet-NeuroD1-ChIP-Seq(GSE30298)/Homer | 1e-488 | -1.124e+03 | 0.0000 | 2110.0 | 29.98% | 4105.0 | 9.75% | motif file (matrix) | svg |
| 8 | T A C G T G C A A G T C C G T A A C G T T G A C A C G T A C T G A C T G G C A T | TCF4(bHLH)/SHSY5Y-TCF4-ChIP-Seq(GSE96915)/Homer | 1e-460 | -1.061e+03 | 0.0000 | 3198.0 | 45.44% | 8804.5 | 20.91% | motif file (matrix) | svg |
| 9 | T G A C A T G C C G T A A T C G A T G C C A G T C A T G A C T G A G T C G T A C | HEB(bHLH)/mES-Heb-ChIP-Seq(GSE53233)/Homer | 1e-438 | -1.010e+03 | 0.0000 | 3096.0 | 43.99% | 8540.9 | 20.28% | motif file (matrix) | svg |
| 10 | C A T G A G T C G A C T C G T A C G A T G C A T G A C T G C A T C G A T C T A G C A T G T G A C | Mef2b(MADS)/HEK293-Mef2b.V5-ChIP-Seq(GSE67450)/Homer | 1e-423 | -9.746e+02 | 0.0000 | 2633.0 | 37.41% | 6627.3 | 15.74% | motif file (matrix) | svg |
| 11 | C T G A T G A C T G A C C G T A A C G T T G A C A G C T C T A G A C G T G A C T | Olig2(bHLH)/Neuron-Olig2-ChIP-Seq(GSE30882)/Homer | 1e-409 | -9.436e+02 | 0.0000 | 3772.0 | 53.59% | 12155.4 | 28.86% | motif file (matrix) | svg |
| 12 | C G A T T G C A T G C A G A T C C G T A A C T G T G A C G A C T C A T G A C T G | Tcf21(bHLH)/ArterySmoothMuscle-Tcf21-ChIP-Seq(GSE61369)/Homer | 1e-368 | -8.492e+02 | 0.0000 | 1969.0 | 27.98% | 4369.2 | 10.37% | motif file (matrix) | svg |
| 13 | C A T G G T A C G A C T G C T A C G T A C G T A C G T A G C T A G A C T C T G A T C A G G T A C | Mef2c(MADS)/GM12878-Mef2c-ChIP-Seq(GSE32465)/Homer | 1e-360 | -8.307e+02 | 0.0000 | 1826.0 | 25.94% | 3887.9 | 9.23% | motif file (matrix) | svg |
| 14 | C G T A T G A C T C G A A G T C C G T A A T C G A T G C A C G T A C T G A G T C | E2A(bHLH)/proBcell-E2A-ChIP-Seq(GSE21978)/Homer | 1e-352 | -8.111e+02 | 0.0000 | 2372.0 | 33.70% | 6133.8 | 14.56% | motif file (matrix) | svg |
| 15 | T A C G T A C G C T A G T C A G A G T C C G T A A T C G A T G C A C G T A C T G A G T C G A C T | Ascl2(bHLH)/ESC-Ascl2-ChIP-Seq(GSE97712)/Homer | 1e-309 | -7.126e+02 | 0.0000 | 1931.0 | 27.44% | 4682.4 | 11.12% | motif file (matrix) | svg |
| 16 | C A T G A G T C A G C T C G T A C G A T C G A T G C A T G C A T C G A T C T G A C A T G T G A C | Mef2d(MADS)/Retina-Mef2d-ChIP-Seq(GSE61391)/Homer | 1e-296 | -6.827e+02 | 0.0000 | 986.0 | 14.01% | 1457.1 | 3.46% | motif file (matrix) | svg |
| 17 | C T G A T C A G G T A C G C T A A C T G T G A C G C A T C A T G | SCL(bHLH)/HPC7-Scl-ChIP-Seq(GSE13511)/Homer | 1e-296 | -6.823e+02 | 0.0000 | 5302.0 | 75.33% | 22778.3 | 54.08% | motif file (matrix) | svg |
| 18 | G T A C G A C T C G T A C T G A T C G A C G T A G C T A C A G T C T G A T A C G | Mef2a(MADS)/HL1-Mef2a.biotin-ChIP-Seq(GSE21529)/Homer | 1e-242 | -5.573e+02 | 0.0000 | 1410.0 | 20.03% | 3201.6 | 7.60% | motif file (matrix) | svg |
| 19 | A C G T T C G A T C G A A G T C G T C A T A C G A T G C A C G T A C T G A G C T | Myf5(bHLH)/GM-Myf5-ChIP-Seq(GSE24852)/Homer | 1e-238 | -5.490e+02 | 0.0000 | 1377.0 | 19.57% | 3105.1 | 7.37% | motif file (matrix) | svg |
| 20 | T C G A A G T C C G T A A T C G A T G C C G A T A C T G A G T C A G C T A C T G | Tcf12(bHLH)/GM12878-Tcf12-ChIP-Seq(GSE32465)/Homer | 1e-236 | -5.447e+02 | 0.0000 | 1562.0 | 22.19% | 3833.2 | 9.10% | motif file (matrix) | svg |
| 21 | G T C A T G C A G C T A A G T C C G T A A C T G T G A C G C A T T C A G C A G T | Ap4(bHLH)/AML-Tfap4-ChIP-Seq(GSE45738)/Homer | 1e-232 | -5.360e+02 | 0.0000 | 2058.0 | 29.24% | 5955.6 | 14.14% | motif file (matrix) | svg |
| 22 | T C G A T C G A A G T C C G T A C T A G T A G C A C G T A C T G | MyoG(bHLH)/C2C12-MyoG-ChIP-Seq(GSE36024)/Homer | 1e-230 | -5.311e+02 | 0.0000 | 1707.0 | 24.25% | 4474.7 | 10.62% | motif file (matrix) | svg |
| 23 | C T G A T C A G A G T C C G T A A T C G A T G C C G A T A C T G A G T C G A C T A T C G A G T C | MyoD(bHLH)/Myotube-MyoD-ChIP-Seq(GSE21614)/Homer | 1e-183 | -4.220e+02 | 0.0000 | 1269.0 | 18.03% | 3150.3 | 7.48% | motif file (matrix) | svg |
| 24 | A C T G C A T G T C G A A C G T A C T G C G T A A T C G A C G T G T A C C G T A G A C T A G T C | Fos(bZIP)/TSC-Fos-ChIP-Seq(GSE110950)/Homer | 1e-142 | -3.286e+02 | 0.0000 | 1244.0 | 17.68% | 3451.7 | 8.20% | motif file (matrix) | svg |
| 25 | A C T G C T A G T C G A C G A T C A T G G C T A A T C G C G A T G T A C G C T A A G C T G T A C | Fra1(bZIP)/BT549-Fra1-ChIP-Seq(GSE46166)/Homer | 1e-140 | -3.244e+02 | 0.0000 | 1183.0 | 16.81% | 3218.0 | 7.64% | motif file (matrix) | svg |
| 26 | A G C T T C G A G T A C T C G A A T G C A T G C G C A T A T C G A G T C A G C T | Snail1(Zf)/LS174T-SNAIL1.HA-ChIP-Seq(GSE127183)/Homer | 1e-136 | -3.142e+02 | 0.0000 | 1324.0 | 18.81% | 3861.2 | 9.17% | motif file (matrix) | svg |
| 27 | C A T G C T A G T C G A A C G T A C T G C G T A T A G C C G A T T G A C C G T A A G C T G A T C | Fra2(bZIP)/Striatum-Fra2-ChIP-Seq(GSE43429)/Homer | 1e-134 | -3.104e+02 | 0.0000 | 992.0 | 14.09% | 2511.2 | 5.96% | motif file (matrix) | svg |
| 28 | C T G A C T A G A T C G A G C T A C T G G A C T A G T C C T G A | Tbx5(T-box)/HL1-Tbx5.biotin-ChIP-Seq(GSE21529)/Homer | 1e-133 | -3.069e+02 | 0.0000 | 3863.0 | 54.89% | 16980.8 | 40.32% | motif file (matrix) | svg |
| 29 | C T A G T C G A A C G T A C T G C G T A A T G C A C G T G T A C C G T A A G C T G A T C G T A C | Atf3(bZIP)/GBM-ATF3-ChIP-Seq(GSE33912)/Homer | 1e-131 | -3.025e+02 | 0.0000 | 1285.0 | 18.26% | 3754.3 | 8.91% | motif file (matrix) | svg |
| 30 | C T A G T C G A G C A T C A T G G C T A T A G C C G A T G T A C C T G A A G C T | JunB(bZIP)/DendriticCells-Junb-ChIP-Seq(GSE36099)/Homer | 1e-130 | -3.011e+02 | 0.0000 | 1120.0 | 15.91% | 3067.5 | 7.28% | motif file (matrix) | svg |
| 31 | A G C T G T C A C G T A A C G T A C G T C T G A T C A G A T G C | Lhx2(Homeobox)/HFSC-Lhx2-ChIP-Seq(GSE48068)/Homer | 1e-130 | -3.000e+02 | 0.0000 | 2491.0 | 35.39% | 9516.9 | 22.60% | motif file (matrix) | svg |
| 32 | A T G C T G C A A G T C C G T A A G T C A G T C A C G T A C T G A T G C G T C A | E2A(bHLH),near\_PU.1/Bcell-PU.1-ChIP-Seq(GSE21512)/Homer | 1e-129 | -2.987e+02 | 0.0000 | 1926.0 | 27.37% | 6708.5 | 15.93% | motif file (matrix) | svg |
| 33 | T C G A A C G T C A T G G C T A T A G C C G A T G T A C G C T A A C G T A T G C | AP-1(bZIP)/ThioMac-PU.1-ChIP-Seq(GSE21512)/Homer | 1e-126 | -2.910e+02 | 0.0000 | 1360.0 | 19.32% | 4142.8 | 9.84% | motif file (matrix) | svg |
| 34 | T G C A C T G A A T G C G T C A A C G T A T G C A C G T A C T G A C T G T G C A | ZBTB18(Zf)/HEK293-ZBTB18.GFP-ChIP-Seq(GSE58341)/Homer | 1e-125 | -2.881e+02 | 0.0000 | 1002.0 | 14.24% | 2646.1 | 6.28% | motif file (matrix) | svg |
| 35 | C A G T T G C A A C G T A C T G C G T A A T C G C G A T T G A C C G T A A C G T | BATF(bZIP)/Th17-BATF-ChIP-Seq(GSE39756)/Homer | 1e-122 | -2.817e+02 | 0.0000 | 1283.0 | 18.23% | 3856.6 | 9.16% | motif file (matrix) | svg |
| 36 | C T A G T C G A C G A T A C T G C G T A T A C G A G C T T G A C G C T A A C G T G A T C T A G C | Fosl2(bZIP)/3T3L1-Fosl2-ChIP-Seq(GSE56872)/Homer | 1e-120 | -2.767e+02 | 0.0000 | 719.0 | 10.22% | 1607.5 | 3.82% | motif file (matrix) | svg |
| 37 | A T G C T A C G A G C T T G C A C G T A C G A T A C G T C T G A | DLX5(Homeobox)/BasalGanglia-Dlx5-ChIP-seq(GSE124936)/Homer | 1e-118 | -2.740e+02 | 0.0000 | 1972.0 | 28.02% | 7111.6 | 16.89% | motif file (matrix) | svg |
| 38 | T C G A C A G T A C T G A G C T C G T A C G T A A C G T A C G T C T G A T A G C | Dlx3(Homeobox)/Kerainocytes-Dlx3-ChIP-Seq(GSE89884)/Homer | 1e-112 | -2.596e+02 | 0.0000 | 1809.0 | 25.70% | 6425.0 | 15.26% | motif file (matrix) | svg |
| 39 | T A C G C T A G T A C G A G T C C G T A A G T C A G T C A C G T A C T G A G T C G A T C T A G C | Slug(Zf)/Mesoderm-Snai2-ChIP-Seq(GSE61475)/Homer | 1e-107 | -2.479e+02 | 0.0000 | 866.0 | 12.30% | 2280.6 | 5.42% | motif file (matrix) | svg |
| 40 | C T A G T C G A A C G T A C T G C G T A T A G C C G A T G T A C C G T A A G C T G A T C G T A C | Jun-AP1(bZIP)/K562-cJun-ChIP-Seq(GSE31477)/Homer | 1e-106 | -2.445e+02 | 0.0000 | 553.0 | 7.86% | 1127.0 | 2.68% | motif file (matrix) | svg |
| 41 | T C A G C T A G A T G C G A C T T G C A C G T A A G C T A C G T C T G A T A C G | En1(Homeobox)/SUM149-EN1-ChIP-Seq(GSE120957)/Homer | 1e-104 | -2.411e+02 | 0.0000 | 3426.0 | 48.68% | 15150.4 | 35.97% | motif file (matrix) | svg |
| 42 | A T C G A T C G A T G C G A C T T C G A C G T A G C A T A G C T C T G A T A C G | DLX2(Homeobox)/BasalGanglia-Dlx2-ChIP-seq(GSE124936)/Homer | 1e-103 | -2.386e+02 | 0.0000 | 3091.0 | 43.92% | 13298.0 | 31.57% | motif file (matrix) | svg |
| 43 | G T A C G A T C C A G T A G T C A G T C A G T C T G C A G A T C C T G A A T G C G T C A A C G T | WT1(Zf)/Kidney-WT1-ChIP-Seq(GSE90016)/Homer | 1e-101 | -2.332e+02 | 0.0000 | 759.0 | 10.78% | 1922.4 | 4.56% | motif file (matrix) | svg |
| 44 | A T C G A T G C A G T C T A G C G A C T T C G A G C T A G C A T A G C T C T G A | DLX1(Homeobox)/BasalGanglia-Dlx1-ChIP-seq(GSE124936)/Homer | 1e-100 | -2.325e+02 | 0.0000 | 2802.0 | 39.81% | 11776.8 | 27.96% | motif file (matrix) | svg |
| 45 | A T G C T A C G A G T C G A C T T G C A C T G A G A C T A C G T C T G A T C A G | LHX9(Homeobox)/Hct116-LHX9.V5-ChIP-Seq(GSE116822)/Homer | 1e-99 | -2.301e+02 | 0.0000 | 2810.0 | 39.93% | 11844.2 | 28.12% | motif file (matrix) | svg |
| 46 | C G T A C A T G A G T C G A C T T G C A C G T A A C G T A C G T C T G A T C A G | Lhx1(Homeobox)/EmbryoCarcinoma-Lhx1-ChIP-Seq(GSE70957)/Homer | 1e-89 | -2.072e+02 | 0.0000 | 2498.0 | 35.49% | 10400.9 | 24.70% | motif file (matrix) | svg |
| 47 | C T A G C T G A T C A G A T C G A G C T C A T G G A C T A G T C C T G A T G C A | Tbx6(T-box)/ESC-Tbx6-ChIP-Seq(GSE93524)/Homer | 1e-88 | -2.040e+02 | 0.0000 | 1578.0 | 22.42% | 5732.9 | 13.61% | motif file (matrix) | svg |
| 48 | T G C A G T A C C G T A A T C G A C T G A C G T C T A G C G A T T C G A A G T C | ZEB1(Zf)/PDAC-ZEB1-ChIP-Seq(GSE64557)/Homer | 1e-88 | -2.037e+02 | 0.0000 | 2160.0 | 30.69% | 8657.6 | 20.56% | motif file (matrix) | svg |
| 49 | G C T A G A C T G A C T T G C A C G T A A G T C C G T A T A G C G A T C G A C T | Eomes(T-box)/H9-Eomes-ChIP-Seq(GSE26097)/Homer | 1e-81 | -1.873e+02 | 0.0000 | 3156.0 | 44.84% | 14236.6 | 33.80% | motif file (matrix) | svg |
| 50 | C T G A C T A G A C T G G C A T C T A G G C A T A T C G C T G A C G T A G T C A | Tbx21(T-box)/GM12878-TBX21-ChIP-Seq(Encode)/Homer | 1e-79 | -1.822e+02 | 0.0000 | 1613.0 | 22.92% | 6085.3 | 14.45% | motif file (matrix) | svg |
| 51 | G A C T C G A T C T G A G T C A G A C T C G A T T C G A C G T A G C T A G C T A T G A C G T A C C G T A A C T G T G C A C G A T A C T G A C G T | Pitx1:Ebox(Homeobox,bHLH)/Hindlimb-Pitx1-ChIP-Seq(GSE41591)/Homer | 1e-75 | -1.738e+02 | 0.0000 | 605.0 | 8.60% | 1576.4 | 3.74% | motif file (matrix) | svg |
| 52 | C T G A C T G A C A T G A T C G A G C T A T C G G A C T C A T G C T G A G T C A | Tbr1(T-box)/Cortex-Tbr1-ChIP-Seq(GSE71384)/Homer | 1e-74 | -1.715e+02 | 0.0000 | 2208.0 | 31.37% | 9238.0 | 21.93% | motif file (matrix) | svg |
| 53 | C A G T T C A G G A T C A C T G A C G T C T A G A C T G A C T G G A C T C T A G | Egr1(Zf)/K562-Egr1-ChIP-Seq(GSE32465)/Homer | 1e-73 | -1.697e+02 | 0.0000 | 769.0 | 10.93% | 2266.8 | 5.38% | motif file (matrix) | svg |
| 54 | C G T A C G T A A G C T G A C T T G C A G T C A A C G T A G C T C T G A T C A G | Lhx3(Homeobox)/Neuron-Lhx3-ChIP-Seq(GSE31456)/Homer | 1e-69 | -1.606e+02 | 0.0000 | 3173.0 | 45.08% | 14670.2 | 34.83% | motif file (matrix) | svg |
| 55 | A C T G C A G T A C G T C G T A C G T A A C G T A C T G C T G A | Nkx6.1(Homeobox)/Islet-Nkx6.1-ChIP-Seq(GSE40975)/Homer | 1e-65 | -1.517e+02 | 0.0000 | 4296.0 | 61.04% | 21407.2 | 50.83% | motif file (matrix) | svg |
| 56 | C G T A C T A G C A T G A G C T A C T G C G A T A T C G C G T A G T C A G T C A | Tbet(T-box)/CD8-Tbet-ChIP-Seq(GSE33802)/Homer | 1e-64 | -1.474e+02 | 0.0000 | 1669.0 | 23.71% | 6695.0 | 15.90% | motif file (matrix) | svg |
| 57 | C T A G A C T G T G C A A G T C C G T A A C T G A C T G A C G T C T A G C G A T T A C G A G T C | ZEB2(Zf)/SNU398-ZEB2-ChIP-Seq(GSE103048)/Homer | 1e-63 | -1.470e+02 | 0.0000 | 1184.0 | 16.82% | 4300.4 | 10.21% | motif file (matrix) | svg |
| 58 | A G T C A C G T A C T G A G C T A C G T A C G T G T C A A G T C | Foxo1(Forkhead)/RAW-Foxo1-ChIP-Seq(Fan\_et\_al.)/Homer | 1e-60 | -1.393e+02 | 0.0000 | 2750.0 | 39.07% | 12575.7 | 29.86% | motif file (matrix) | svg |
| 59 | A G C T C A T G G C A T G A T C T G C A C T A G G A T C A C G T | Tgif2(Homeobox)/mES-Tgif2-ChIP-Seq(GSE55404)/Homer | 1e-51 | -1.181e+02 | 0.0000 | 3937.0 | 55.94% | 19761.9 | 46.92% | motif file (matrix) | svg |
| 60 | C G A T C T A G A C G T G T C A C G T A C G T A A G T C C G T A | Foxo3(Forkhead)/U2OS-Foxo3-ChIP-Seq(E-MTAB-2701)/Homer | 1e-46 | -1.079e+02 | 0.0000 | 1548.0 | 21.99% | 6506.9 | 15.45% | motif file (matrix) | svg |
| 61 | C G A T T A C G T G A C G A C T C A T G C G T A T A C G A C G T G T A C C T G A | Bach2(bZIP)/OCILy7-Bach2-ChIP-Seq(GSE44420)/Homer | 1e-41 | -9.626e+01 | 0.0000 | 349.0 | 4.96% | 923.3 | 2.19% | motif file (matrix) | svg |
| 62 | C T A G T C A G C A G T T C A G A C T G A C T G G A T C C T A G A C T G C T A G T C A G A T G C | KLF14(Zf)/HEK293-KLF14.GFP-ChIP-Seq(GSE58341)/Homer | 1e-40 | -9.324e+01 | 0.0000 | 1290.0 | 18.33% | 5352.1 | 12.71% | motif file (matrix) | svg |
| 63 | A G C T T G A C C G A T C G A T C T A G A C G T C A G T C A G T G C T A A G T C | FOXK1(Forkhead)/HEK293-FOXK1-ChIP-Seq(GSE51673)/Homer | 1e-39 | -9.188e+01 | 0.0000 | 2008.0 | 28.53% | 9167.8 | 21.77% | motif file (matrix) | svg |
| 64 | G C A T G C A T C T G A A C G T C T G A A C G T C G T A C G T A C G T A A G T C G T C A G T C A | Foxf1(Forkhead)/Lung-Foxf1-ChIP-Seq(GSE77951)/Homer | 1e-36 | -8.364e+01 | 0.0000 | 2117.0 | 30.08% | 9892.4 | 23.49% | motif file (matrix) | svg |
| 65 | G A C T G C A T C T A G C G A T G A T C T C G A C A T G G A T C | Tgif1(Homeobox)/mES-Tgif1-ChIP-Seq(GSE55404)/Homer | 1e-33 | -7.802e+01 | 0.0000 | 3697.0 | 52.53% | 19059.7 | 45.26% | motif file (matrix) | svg |
| 66 | A G C T A G T C A G T C A C G T C T A G A C G T A C G T A C G T C G T A A G T C G A T C C G T A | FOXP1(Forkhead)/H9-FOXP1-ChIP-Seq(GSE31006)/Homer | 1e-32 | -7.451e+01 | 0.0000 | 954.0 | 13.55% | 3868.3 | 9.18% | motif file (matrix) | svg |
| 67 | C G T A G C T A C G A T C T A G A C G T G T C A C G T A C G T A A G T C C G T A T G C A T A C G | FoxL2(Forkhead)/Ovary-FoxL2-ChIP-Seq(GSE60858)/Homer | 1e-29 | -6.680e+01 | 0.0000 | 1871.0 | 26.58% | 8823.3 | 20.95% | motif file (matrix) | svg |
| 68 | A G T C C G A T C T G A C G T A A C G T C A G T T C A G T G A C | Isl1(Homeobox)/Neuron-Isl1-ChIP-Seq(GSE31456)/Homer | 1e-27 | -6.429e+01 | 0.0000 | 2851.0 | 40.51% | 14394.1 | 34.18% | motif file (matrix) | svg |
| 69 | T C A G T A C G T A G C A C G T A C T G C G A T A G T C C G T A T A C G A G T C | Meis1(Homeobox)/MastCells-Meis1-ChIP-Seq(GSE48085)/Homer | 1e-26 | -6.046e+01 | 0.0000 | 1909.0 | 27.12% | 9147.2 | 21.72% | motif file (matrix) | svg |
| 70 | C A T G A C T G C T A G T C G A T C G A T C G A T C G A T C A G T C A G T C A G T G A C T G A C C G T A A C T G T G C A C G A T A C T G | RBPJ:Ebox(?,bHLH)/Panc1-Rbpj1-ChIP-Seq(GSE47459)/Homer | 1e-25 | -5.912e+01 | 0.0000 | 436.0 | 6.19% | 1520.6 | 3.61% | motif file (matrix) | svg |
| 71 | T A C G T A G C G C T A C G A T C T A G A C G T C A G T C A G T G C T A A G T C G T C A G C A T | FOXK2(Forkhead)/U2OS-FOXK2-ChIP-Seq(E-MTAB-2204)/Homer | 1e-23 | -5.525e+01 | 0.0000 | 1107.0 | 15.73% | 4904.1 | 11.64% | motif file (matrix) | svg |
| 72 | A T G C G A C T A C G T C T A G A C G T A C G T A C G T C T G A G A T C G C T A A G C T C G T A | Foxa2(Forkhead)/Liver-Foxa2-ChIP-Seq(GSE25694)/Homer | 1e-23 | -5.305e+01 | 0.0000 | 1453.0 | 20.65% | 6786.4 | 16.11% | motif file (matrix) | svg |
| 73 | T A C G T C G A G A C T A C T G C T G A A G T C T C A G G A C T T G A C C T G A | Atf1(bZIP)/K562-ATF1-ChIP-Seq(GSE31477)/Homer | 1e-22 | -5.147e+01 | 0.0000 | 824.0 | 11.71% | 3492.5 | 8.29% | motif file (matrix) | svg |
| 74 | A C G T T A C G G A T C A C T G A C G T C T A G A C T G A C T G G A T C C T A G C A T G C T A G | Egr2(Zf)/Thymocytes-Egr2-ChIP-Seq(GSE34254)/Homer | 1e-22 | -5.112e+01 | 0.0000 | 188.0 | 2.67% | 503.8 | 1.20% | motif file (matrix) | svg |
| 75 | C T G A T C A G C A G T C T A G A C T G C T A G G A T C A T C G A C T G C T G A T C A G G A T C | Sp5(Zf)/mES-Sp5.Flag-ChIP-Seq(GSE72989)/Homer | 1e-21 | -5.037e+01 | 0.0000 | 634.0 | 9.01% | 2550.5 | 6.06% | motif file (matrix) | svg |
| 76 | T G C A T C G A T C G A A C G T C A T G C G T A A G T C T C A G A C G T G T A C C G T A A G C T | CREB5(bZIP)/LNCaP-CREB5.V5-ChIP-Seq(GSE137775)/Homer | 1e-21 | -5.024e+01 | 0.0000 | 578.0 | 8.21% | 2276.9 | 5.41% | motif file (matrix) | svg |
| 77 | T C G A A C G T A C G T C T G A G A T C T C A G G A C T G T C A C G T A A G C T G T C A C T A G A G C T A C G T T C G A | NFIL3(bZIP)/HepG2-NFIL3-ChIP-Seq(Encode)/Homer | 1e-21 | -4.980e+01 | 0.0000 | 1110.0 | 15.77% | 5004.0 | 11.88% | motif file (matrix) | svg |
| 78 | T A C G T C G A C A G T A C T G G C T A A T G C C G A T G T A C C G T A A C T G T A G C C G T A | NF-E2(bZIP)/K562-NFE2-ChIP-Seq(GSE31477)/Homer | 1e-18 | -4.279e+01 | 0.0000 | 117.0 | 1.66% | 270.5 | 0.64% | motif file (matrix) | svg |
| 79 | G A C T C A G T A G C T C G A T A G T C G A T C A G T C C G T A A T G C T C A G | Rbpj1(?)/Panc1-Rbpj1-ChIP-Seq(GSE47459)/Homer | 1e-18 | -4.218e+01 | 0.0000 | 1657.0 | 23.54% | 8117.6 | 19.27% | motif file (matrix) | svg |
| 80 | G A C T C T A G A T G C A G T C G T C A T A C G A T G C A T C G | HIC1(Zf)/Treg-ZBTB29-ChIP-Seq(GSE99889)/Homer | 1e-17 | -4.124e+01 | 0.0000 | 1851.0 | 26.30% | 9220.4 | 21.89% | motif file (matrix) | svg |
| 81 | G T C A G C A T A C T G G T A C G A C T A C T G G C T A A T C G C A G T G T A C C G T A A G C T | Nrf2(bZIP)/Lymphoblast-Nrf2-ChIP-Seq(GSE37589)/Homer | 1e-17 | -4.077e+01 | 0.0000 | 100.0 | 1.42% | 217.6 | 0.52% | motif file (matrix) | svg |
| 82 | C G T A C G T A C G T A G C A T G C A T T A C G G T A C G A C T A C T G C G T A A T C G A C G T G T A C C G T A A G C T | Bach1(bZIP)/K562-Bach1-ChIP-Seq(GSE31477)/Homer | 1e-17 | -4.022e+01 | 0.0000 | 123.0 | 1.75% | 302.8 | 0.72% | motif file (matrix) | svg |
| 83 | A G C T A C G T A C T G A T G C A G T C C G T A C T G A T A C G | NF1-halfsite(CTF)/LNCaP-NF1-ChIP-Seq(Unpublished)/Homer | 1e-17 | -3.942e+01 | 0.0000 | 1684.0 | 23.93% | 8328.8 | 19.78% | motif file (matrix) | svg |
| 84 | T C G A A C T G A C T G C G T A C G T A T C G A A G T C C T G A A T C G G T A C G C A T C A T G | ETS:E-box(ETS,bHLH)/HPC7-Scl-ChIP-Seq(GSE22178)/Homer | 1e-17 | -3.934e+01 | 0.0000 | 157.0 | 2.23% | 438.3 | 1.04% | motif file (matrix) | svg |
| 85 | C T A G A C T G C T A G T C A G T C A G T A C G C T A G A C T G | Maz(Zf)/HepG2-Maz-ChIP-Seq(GSE31477)/Homer | 1e-16 | -3.887e+01 | 0.0000 | 860.0 | 12.22% | 3862.8 | 9.17% | motif file (matrix) | svg |
| 86 | G C T A C G T A A G T C A C G T T C G A T A C G A C T G A G C T A G T C T C G A | RORgt(NR)/EL4-RORgt.Flag-ChIP-Seq(GSE56019)/Homer | 1e-16 | -3.874e+01 | 0.0000 | 212.0 | 3.01% | 671.9 | 1.60% | motif file (matrix) | svg |
| 87 | G C T A C G T A A G T C A C G T T C G A T A C G A C T G A G C T A G T C T C G A | RORgt(NR)/EL4-RORgt.Flag-ChIP-Seq(GSE56019)/Homer | 1e-16 | -3.874e+01 | 0.0000 | 212.0 | 3.01% | 671.9 | 1.60% | motif file (matrix) | svg |
| 88 | G C T A T C G A C G T A C T A G A G C T G T C A G T C A C G T A A G T C C G T A | FOXA1(Forkhead)/LNCAP-FOXA1-ChIP-Seq(GSE27824)/Homer | 1e-16 | -3.834e+01 | 0.0000 | 2333.0 | 33.15% | 12025.8 | 28.55% | motif file (matrix) | svg |
| 89 | T A G C C T A G T C G A G A C T A C T G C T G A A G T C T C A G G C A T T G A C C T G A A G C T | Atf7(bZIP)/3T3L1-Atf7-ChIP-Seq(GSE56872)/Homer | 1e-16 | -3.823e+01 | 0.0000 | 555.0 | 7.89% | 2302.1 | 5.47% | motif file (matrix) | svg |
| 90 | C G T A C G T A C G T A A G T C A G C T C T G A A C T G C T A G A G C T A G T C C G T A T C A G | RORg(NR)/Liver-Rorc-ChIP-Seq(GSE101115)/Homer | 1e-16 | -3.748e+01 | 0.0000 | 176.0 | 2.50% | 526.6 | 1.25% | motif file (matrix) | svg |
| 91 | T A G C G T A C A G T C G T A C C G A T A G T C A G T C A G T C A G T C A G T C C G T A G A T C | Zfp281(Zf)/ES-Zfp281-ChIP-Seq(GSE81042)/Homer | 1e-15 | -3.656e+01 | 0.0000 | 216.0 | 3.07% | 703.7 | 1.67% | motif file (matrix) | svg |
| 92 | C T G A C G A T C T A G T C A G G A T C C T G A T C A G G A T C C T G A A C T G A G T C G C T A A C G T A G T C G C A T | PRDM9(Zf)/Testis-DMC1-ChIP-Seq(GSE35498)/Homer | 1e-15 | -3.559e+01 | 0.0000 | 494.0 | 7.02% | 2030.2 | 4.82% | motif file (matrix) | svg |
| 93 | A T G C C T G A A T C G T A C G A G T C C G A T T C A G C G A T C T A G A G C T G T C A G T C A C G T A A G T C C G T A T A C G C T G A | Fox:Ebox(Forkhead,bHLH)/Panc1-Foxa2-ChIP-Seq(GSE47459)/Homer | 1e-15 | -3.527e+01 | 0.0000 | 1550.0 | 22.02% | 7677.6 | 18.23% | motif file (matrix) | svg |
| 94 | G A C T G T A C T G C A A C G T G A T C G C T A T C G A A C G T A G T C C G T A | Pdx1(Homeobox)/Islet-Pdx1-ChIP-Seq(SRA008281)/Homer | 1e-14 | -3.451e+01 | 0.0000 | 1596.0 | 22.68% | 7951.1 | 18.88% | motif file (matrix) | svg |
| 95 | A T G C T C A G T C G A G C A T A C T G C G T A A G T C T C A G G A C T T G A C C G T A A G C T | Atf2(bZIP)/3T3L1-Atf2-ChIP-Seq(GSE56872)/Homer | 1e-14 | -3.364e+01 | 0.0000 | 375.0 | 5.33% | 1467.4 | 3.48% | motif file (matrix) | svg |
| 96 | C G T A C G T A C G T A A G T C A G C T C T G A A C T G A C T G A G C T A G T C C G T A C T A G C T A G C T A G T G C A | RORa(NR)/Liver-Rora-ChIP-Seq(GSE101115)/Homer | 1e-14 | -3.352e+01 | 0.0000 | 225.0 | 3.20% | 765.9 | 1.82% | motif file (matrix) | svg |
| 97 | A G T C G C A T C G T A C G T A G T A C A C G T A C T G G A T C G A T C T C G A | BMYB(HTH)/Hela-BMYB-ChIP-Seq(GSE27030)/Homer | 1e-14 | -3.305e+01 | 0.0000 | 1857.0 | 26.39% | 9454.2 | 22.45% | motif file (matrix) | svg |
| 98 | T G C A A G C T C A T G C G T A A G C T A C T G G A T C G T C A C G T A A G C T | Atf4(bZIP)/MEF-Atf4-ChIP-Seq(GSE35681)/Homer | 1e-14 | -3.286e+01 | 0.0000 | 434.0 | 6.17% | 1765.7 | 4.19% | motif file (matrix) | svg |
| 99 | C T A G T C G A C G A T C T A G G C A T C A G T C T A G G A T C C G T A G T C A | CEBP:AP1(bZIP)/ThioMac-CEBPb-ChIP-Seq(GSE21512)/Homer | 1e-14 | -3.285e+01 | 0.0000 | 1058.0 | 15.03% | 5027.7 | 11.94% | motif file (matrix) | svg |
| 100 | A G T C G A C T C A G T A C T G C T A G T G A C G C T A A T G C G C A T A T C G C G A T A C T G G A T C G T A C G T C A C T G A | NF1(CTF)/LNCAP-NF1-ChIP-Seq(Unpublished)/Homer | 1e-14 | -3.274e+01 | 0.0000 | 338.0 | 4.80% | 1299.0 | 3.08% | motif file (matrix) | svg |
| 101 | T A G C T A G C G A C T C T A G A G C T A G T C G T C A T G C A A C G T A T G C G C T A T G C A | Pbx3(Homeobox)/GM12878-PBX3-ChIP-Seq(GSE32465)/Homer | 1e-14 | -3.264e+01 | 0.0000 | 320.0 | 4.55% | 1213.4 | 2.88% | motif file (matrix) | svg |
| 102 | T C G A G C A T A C T G C T G A A G T C T C A G G A C T G T A C C G T A A G C T A G T C G A T C | c-Jun-CRE(bZIP)/K562-cJun-ChIP-Seq(GSE31477)/Homer | 1e-13 | -2.996e+01 | 0.0000 | 356.0 | 5.06% | 1415.1 | 3.36% | motif file (matrix) | svg |
| 103 | T C A G G A C T C A G T C T G A A G C T C T A G G A C T T G C A C T G A A G T C | HLF(bZIP)/HSC-HLF.Flag-ChIP-Seq(GSE69817)/Homer | 1e-12 | -2.906e+01 | 0.0000 | 1377.0 | 19.57% | 6863.3 | 16.30% | motif file (matrix) | svg |
| 104 | T C G A A C G T A C T G C T G A A G T C T C A G A G C T G T A C C G T A A G C T G A T C T C G A | JunD(bZIP)/K562-JunD-ChIP-Seq/Homer | 1e-12 | -2.812e+01 | 0.0000 | 113.0 | 1.61% | 319.5 | 0.76% | motif file (matrix) | svg |
| 105 | C T A G T A C G G A T C G T A C G C T A A G C T A G C T G T C A T C G A T A G C | Nanog(Homeobox)/mES-Nanog-ChIP-Seq(GSE11724)/Homer | 1e-11 | -2.580e+01 | 0.0000 | 4714.0 | 66.98% | 26577.3 | 63.11% | motif file (matrix) | svg |
| 106 | T A C G A T G C G A C T A C T G A G C T A G T C G T C A T G C A A C G T A G T C G C T A T G C A | Pknox1(Homeobox)/ES-Prep1-ChIP-Seq(GSE63282)/Homer | 1e-10 | -2.511e+01 | 0.0000 | 287.0 | 4.08% | 1133.0 | 2.69% | motif file (matrix) | svg |
| 107 | G A C T C T A G G A T C C A G T A C T G C T G A A T G C G C A T A T G C C T G A | MafA(bZIP)/Islet-MafA-ChIP-Seq(GSE30298)/Homer | 1e-10 | -2.435e+01 | 0.0000 | 911.0 | 12.94% | 4408.2 | 10.47% | motif file (matrix) | svg |
| 108 | C T A G T A G C A G T C G T C A C T G A A C G T C G T A C G T A C G T A G C T A | Hoxd13(Homeobox)/ChickenMSG-Hoxd13.Flag-ChIP-Seq(GSE86088)/Homer | 1e-10 | -2.377e+01 | 0.0000 | 2722.0 | 38.68% | 14724.8 | 34.96% | motif file (matrix) | svg |
| 109 | A G T C A T C G C T A G A G C T G A C T C T A G A G T C A G T C G C T A C A G T T C A G T C A G G A T C C T G A T C G A G A T C | RFX(HTH)/K562-RFX3-ChIP-Seq(SRA012198)/Homer | 1e-10 | -2.341e+01 | 0.0000 | 95.0 | 1.35% | 271.6 | 0.64% | motif file (matrix) | svg |
| 110 | A G T C T A G C G A C T A C G T C T A G A C G T A C G T A C G T C T G A A G T C G C T A G A C T C G T A C T A G A C T G | Foxa3(Forkhead)/Liver-Foxa3-ChIP-Seq(GSE77670)/Homer | 1e-10 | -2.336e+01 | 0.0000 | 626.0 | 8.89% | 2895.4 | 6.87% | motif file (matrix) | svg |
| 111 | T C G A G C A T A C G T C T A G G T A C T C G A G C A T T G A C T C G A A C G T | Chop(bZIP)/MEF-Chop-ChIP-Seq(GSE35681)/Homer | 1e-10 | -2.325e+01 | 0.0000 | 333.0 | 4.73% | 1382.6 | 3.28% | motif file (matrix) | svg |
| 112 | A T G C G T A C C G T A A G C T G C A T T A C G A G C T A G C T A G T C A G C T | Sox6(HMG)/Myotubes-Sox6-ChIP-Seq(GSE32627)/Homer | 1e-9 | -2.236e+01 | 0.0000 | 2205.0 | 31.33% | 11766.6 | 27.94% | motif file (matrix) | svg |
| 113 | C G A T C T A G A C G T A C G T A C G T C G T A A G C T C G A T A G C T C G T A C T A G T A G C | FoxD3(forkhead)/ZebrafishEmbryo-Foxd3.biotin-ChIP-seq(GSE106676)/Homer | 1e-9 | -2.165e+01 | 0.0000 | 1573.0 | 22.35% | 8168.0 | 19.39% | motif file (matrix) | svg |
| 114 | T G A C G T A C C G T A A C T G T G A C C G A T A C T G A T C G A G C T T A C G T C G A T A G C G T A C C G T A A T C G T G A C G C A T A C T G A C T G A T G C | Twist(bHLH)/HMLE-TWIST1-ChIP-Seq(Chang\_et\_al)/Homer | 1e-9 | -2.139e+01 | 0.0000 | 164.0 | 2.33% | 586.4 | 1.39% | motif file (matrix) | svg |
| 115 | T C A G A G C T A C G T A C G T G T A C G A T C C G T A C T A G C A T G G T C A C G T A T C G A | STAT4(Stat)/CD4-Stat4-ChIP-Seq(GSE22104)/Homer | 1e-9 | -2.104e+01 | 0.0000 | 1228.0 | 17.45% | 6242.9 | 14.82% | motif file (matrix) | svg |
| 116 | C G T A C G A T G A C T G A C T T G A C C T G A A T G C C T G A T A G C A G T C A C G T T C G A C A T G T A C G G A C T A T C G G A C T A C G T C T G A T C G A C G T A | Brachyury(T-box)/Mesoendoderm-Brachyury-ChIP-exo(GSE54963)/Homer | 1e-9 | -2.094e+01 | 0.0000 | 405.0 | 5.75% | 1780.8 | 4.23% | motif file (matrix) | svg |
| 117 | T C G A C T G A T A G C T G A C T C A G T C A G C G T A C G T A T C A G A G C T | ETV1(ETS)/GIST48-ETV1-ChIP-Seq(GSE22441)/Homer | 1e-9 | -2.084e+01 | 0.0000 | 1314.0 | 18.67% | 6730.6 | 15.98% | motif file (matrix) | svg |
| 118 | C T A G A G C T G A C T C A T G A G T C A G T C G T C A C A G T C T A G T C A G G T A C C T G A T C G A G A T C T G A C | Rfx2(HTH)/LoVo-RFX2-ChIP-Seq(GSE49402)/Homer | 1e-9 | -2.073e+01 | 0.0000 | 104.0 | 1.48% | 324.9 | 0.77% | motif file (matrix) | svg |
| 119 | G C T A T C G A C G T A C T A G A G C T G T C A G T C A C G T A A G T C C G T A | FOXA1(Forkhead)/MCF7-FOXA1-ChIP-Seq(GSE26831)/Homer | 1e-8 | -2.061e+01 | 0.0000 | 1962.0 | 27.88% | 10425.8 | 24.76% | motif file (matrix) | svg |
| 120 | T C G A T A G C G T C A A C T G A C T G C G T A C G T A C T A G A G C T T C A G | ERG(ETS)/VCaP-ERG-ChIP-Seq(GSE14097)/Homer | 1e-8 | -2.059e+01 | 0.0000 | 1631.0 | 23.17% | 8531.7 | 20.26% | motif file (matrix) | svg |
| 121 | C T G A C G A T C T A G C G T A A G C T C G A T C A G T C T G A G A C T C T A G C T A G A T G C | PBX2(Homeobox)/K562-PBX2-ChIP-Seq(Encode)/Homer | 1e-8 | -1.994e+01 | 0.0000 | 1340.0 | 19.04% | 6904.0 | 16.39% | motif file (matrix) | svg |
| 122 | C T A G T A C G G A T C G T A C C T G A A G C T T G C A G C T A C G T A G C A T G A T C G C T A | Hoxc9(Homeobox)/Ainv15-Hoxc9-ChIP-Seq(GSE21812)/Homer | 1e-8 | -1.980e+01 | 0.0000 | 862.0 | 12.25% | 4246.3 | 10.08% | motif file (matrix) | svg |
| 123 | C G T A T A G C T A G C T G C A A C T G C T A G C G T A C G T A T C A G G A C T | EHF(ETS)/LoVo-EHF-ChIP-Seq(GSE49402)/Homer | 1e-8 | -1.949e+01 | 0.0000 | 1436.0 | 20.40% | 7459.9 | 17.71% | motif file (matrix) | svg |
| 124 | T C A G A T C G G A C T A C T G G A C T C A G T C T A G C G T A G T A C C G T A C T A G A T C G | Tbx20(T-box)/Heart-Tbx20-ChIP-Seq(GSE29636)/Homer | 1e-8 | -1.907e+01 | 0.0000 | 249.0 | 3.54% | 1017.2 | 2.42% | motif file (matrix) | svg |
| 125 | A G T C C T A G C T A G A G T C G A T C G T A C A G T C C T A G A G T C A G T C A G T C G T A C | Sp2(Zf)/HEK293-Sp2.eGFP-ChIP-Seq(Encode)/Homer | 1e-8 | -1.905e+01 | 0.0000 | 845.0 | 12.01% | 4171.1 | 9.90% | motif file (matrix) | svg |
| 126 | C G T A C T G A C T A G C G T A C G T A A G T C C G T A C A G T G C A T G T C A C G A T A C T G A C G T G C A T G A T C | PGR(NR)/EndoStromal-PGR-ChIP-Seq(GSE69539)/Homer | 1e-7 | -1.827e+01 | 0.0000 | 373.0 | 5.30% | 1657.2 | 3.93% | motif file (matrix) | svg |
| 127 | C A T G T A C G T A G C G A T C G A T C A T G C G T A C G A C T T C A G A T G C C G A T A T C G C A G T A C T G G T A C | Zic3(Zf)/mES-Zic3-ChIP-Seq(GSE37889)/Homer | 1e-7 | -1.826e+01 | 0.0000 | 388.0 | 5.51% | 1735.9 | 4.12% | motif file (matrix) | svg |
| 128 | C G T A C G T A C G T A G C A T G C A T A C T G G T A C G A C T C T A G G C T A T A C G G A C T T G A C C G T A A G C T | NFE2L2(bZIP)/HepG2-NFE2L2-ChIP-Seq(Encode)/Homer | 1e-7 | -1.664e+01 | 0.0000 | 140.0 | 1.99% | 517.8 | 1.23% | motif file (matrix) | svg |
| 129 | T A G C G C A T A G T C G A T C A T G C G A C T C T A G A C T G A C T G C T G A A C T G C T A G A G T C T G A C C G A T | GLIS3(Zf)/Thyroid-Glis3.GFP-ChIP-Seq(GSE103297)/Homer | 1e-7 | -1.661e+01 | 0.0000 | 875.0 | 12.43% | 4400.2 | 10.45% | motif file (matrix) | svg |
| 130 | C G A T C T A G T C G A A G C T C G A T C T G A C G T A A G C T A C T G C T A G A T G C G A T C | Hoxb4(Homeobox)/ES-Hoxb4-ChIP-Seq(GSE34014)/Homer | 1e-7 | -1.631e+01 | 0.0000 | 309.0 | 4.39% | 1358.1 | 3.22% | motif file (matrix) | svg |
| 131 | G A T C G C T A C A G T A C G T T A C G A G T C A T G C C T A G A G T C T C G A | Zfp57(Zf)/H1-ZFP57.HA-ChIP-Seq(GSE115387)/Homer | 1e-6 | -1.606e+01 | 0.0000 | 162.0 | 2.30% | 627.9 | 1.49% | motif file (matrix) | svg |
| 132 | A T G C T C G A T A C G A C G T A T G C A G T C A C G T A G T C A G T C G A T C | Znf263(Zf)/K562-Znf263-ChIP-Seq(GSE31477)/Homer | 1e-6 | -1.594e+01 | 0.0000 | 1367.0 | 19.42% | 7185.2 | 17.06% | motif file (matrix) | svg |
| 133 | A T G C A T C G T A C G A G C T A T C G C T G A A G T C C T A G A G C T A T G C C T G A A T G C | CRE(bZIP)/Promoter/Homer | 1e-6 | -1.560e+01 | 0.0000 | 181.0 | 2.57% | 724.1 | 1.72% | motif file (matrix) | svg |
| 134 | A G T C C T G A A G T C C G A T C A G T G A T C A T G C A C T G A T C G G A C T | Fli1(ETS)/CD8-FLI-ChIP-Seq(GSE20898)/Homer | 1e-6 | -1.553e+01 | 0.0000 | 1018.0 | 14.46% | 5228.5 | 12.41% | motif file (matrix) | svg |
| 135 | T G A C G C T A T G A C C G T A T C A G G A T C C G T A C A T G C A T G C T A G C T A G C T A G | Unknown-ESC-element(?)/mES-Nanog-ChIP-Seq(GSE11724)/Homer | 1e-6 | -1.543e+01 | 0.0000 | 489.0 | 6.95% | 2321.5 | 5.51% | motif file (matrix) | svg |
| 136 | C T G A A G T C C G A T A G C T A T G C G T A C A C G T A T C G C A G T G C A T | Elf4(ETS)/BMDM-Elf4-ChIP-Seq(GSE88699)/Homer | 1e-6 | -1.523e+01 | 0.0000 | 989.0 | 14.05% | 5075.1 | 12.05% | motif file (matrix) | svg |
| 137 | A T G C A G T C C T G A A G T C C G A T A C G T A G T C A G T C A C G T A T C G G A C T A C G T | Etv2(ETS)/ES-ER71-ChIP-Seq(GSE59402)/Homer | 1e-6 | -1.518e+01 | 0.0000 | 960.0 | 13.64% | 4914.0 | 11.67% | motif file (matrix) | svg |
| 138 | A G T C C G A T A C T G A T C G T G A C G C T A C A T G A T C G T G A C C G A T A C T G T A G C G T A C G T C A | Tlx?(NR)/NPC-H3K4me1-ChIP-Seq(GSE16256)/Homer | 1e-6 | -1.477e+01 | 0.0000 | 360.0 | 5.12% | 1649.7 | 3.92% | motif file (matrix) | svg |
| 139 | C T G A C T G A C T A G T C G A C G T A A T G C C G T A A C T G C G T A A C G T C T G A C G A T A G C T C G T A A C G T A G T C C G A T T A C G G T C A G C A T | GATA(Zf),IR3/iTreg-Gata3-ChIP-Seq(GSE20898)/Homer | 1e-6 | -1.451e+01 | 0.0000 | 247.0 | 3.51% | 1068.6 | 2.54% | motif file (matrix) | svg |
| 140 | T C A G T G A C G T A C T G C A G T A C C T A G G T A C A T G C A G T C G T C A A G T C G A C T | Klf9(Zf)/GBM-Klf9-ChIP-Seq(GSE62211)/Homer | 1e-6 | -1.437e+01 | 0.0000 | 256.0 | 3.64% | 1116.4 | 2.65% | motif file (matrix) | svg |
| 141 | T C A G G C T A T C A G C A G T T G A C G T C A A G T C A T C G T G C A G T A C C A G T G A T C | Npas4(bHLH)/Neuron-Npas4-ChIP-Seq(GSE127793)/Homer | 1e-5 | -1.348e+01 | 0.0000 | 637.0 | 9.05% | 3172.0 | 7.53% | motif file (matrix) | svg |
| 142 | C G T A T G A C T A G C T G C A A C T G A C T G C G T A C G T A T C A G G A C T | ELF3(ETS)/PDAC-ELF3-ChIP-Seq(GSE64557)/Homer | 1e-5 | -1.337e+01 | 0.0000 | 849.0 | 12.06% | 4350.0 | 10.33% | motif file (matrix) | svg |
| 143 | C T A G A T G C A T G C C G A T A C T G G A C T A T G C G C T A T G A C A G C T T A G C G C T A | PBX1(Homeobox)/MCF7-PBX1-ChIP-Seq(GSE28007)/Homer | 1e-5 | -1.264e+01 | 0.0000 | 103.0 | 1.46% | 381.5 | 0.91% | motif file (matrix) | svg |
| 144 | C A T G C T A G A G C T G A C T C A T G A G T C G A T C G C T A C G A T C T A G T C A G G T A C C T G A T C G A | X-box(HTH)/NPC-H3K4me1-ChIP-Seq(GSE16256)/Homer | 1e-5 | -1.250e+01 | 0.0000 | 136.0 | 1.93% | 540.2 | 1.28% | motif file (matrix) | svg |
| 145 | C T A G A C G T A G T C C G T A A C T G A G T C G C A T A C T G G C A T A G T C G A C T G A T C G C A T A G T C A G C T | ZNF317(Zf)/HEK293-ZNF317.GFP-ChIP-Seq(GSE58341)/Homer | 1e-5 | -1.228e+01 | 0.0000 | 101.0 | 1.44% | 375.6 | 0.89% | motif file (matrix) | svg |
| 146 | A T G C A G T C G C T A C G A T C G T A G C A T G C T A C G A T C T A G C A T G T G A C G T C A | CArG(MADS)/PUER-Srf-ChIP-Seq(Sullivan\_et\_al.)/Homer | 1e-5 | -1.206e+01 | 0.0000 | 379.0 | 5.39% | 1803.9 | 4.28% | motif file (matrix) | svg |
| 147 | G A C T C A G T G A T C G A T C A C G T G A T C C T G A T A C G C G T A G T C A | STAT6(Stat)/Macrophage-Stat6-ChIP-Seq(GSE38377)/Homer | 1e-5 | -1.196e+01 | 0.0000 | 706.0 | 10.03% | 3595.7 | 8.54% | motif file (matrix) | svg |
| 148 | T C G A T A G C T G C A A C T G A C T G C G T A C G T A C T A G G A C T T A C G | ETS1(ETS)/Jurkat-ETS1-ChIP-Seq(GSE17954)/Homer | 1e-5 | -1.178e+01 | 0.0000 | 1017.0 | 14.45% | 5347.4 | 12.70% | motif file (matrix) | svg |
| 149 | T G C A C T G A A G T C G T C A A C T G A C T G C G T A C G T A C T G A A G C T | EWS:FLI1-fusion(ETS)/SK\_N\_MC-EWS:FLI1-ChIP-Seq(SRA014231)/Homer | 1e-4 | -1.151e+01 | 0.0000 | 589.0 | 8.37% | 2960.5 | 7.03% | motif file (matrix) | svg |
| 150 | T C G A A G C T A C G T A C G T A G T C A G T C A C G T A T C G G A C T A T C G | EWS:ERG-fusion(ETS)/CADO\_ES1-EWS:ERG-ChIP-Seq(SRA014231)/Homer | 1e-4 | -1.134e+01 | 0.0000 | 888.0 | 12.62% | 4635.0 | 11.01% | motif file (matrix) | svg |
| 151 | C G T A A C G T A C T G G T A C C G T A A C G T C G T A C G T A A C G T A C T G A G T C C G T A A C G T C T G A G C A T | OCT:OCT-short(POU,Homeobox)/NPC-OCT6-ChIP-Seq(GSE43916)/Homer | 1e-4 | -1.129e+01 | 0.0000 | 1223.0 | 17.38% | 6538.6 | 15.53% | motif file (matrix) | svg |
| 152 | C A G T C G A T G C A T G C A T G T C A A G C T C A T G C T A G A T G C G T A C | Hoxa11(Homeobox)/ChickenMSG-Hoxa11.Flag-ChIP-Seq(GSE86088)/Homer | 1e-4 | -1.126e+01 | 0.0000 | 3525.0 | 50.09% | 20035.4 | 47.57% | motif file (matrix) | svg |
| 153 | C T G A T A G C T G A C T C A G C T A G G T C A C G T A T C A G A G C T T C A G | ETV4(ETS)/HepG2-ETV4-ChIP-Seq(ENCODE)/Homer | 1e-4 | -1.084e+01 | 0.0001 | 915.0 | 13.00% | 4804.3 | 11.41% | motif file (matrix) | svg |
| 154 | C T G A C T A G A T C G G C A T A C T G G T A C A T G C C G T A A C T G G C T A A G T C C G T A | Tbox:Smad(T-box,MAD)/ESCd5-Smad2\_3-ChIP-Seq(GSE29422)/Homer | 1e-4 | -1.073e+01 | 0.0001 | 206.0 | 2.93% | 916.3 | 2.18% | motif file (matrix) | svg |
| 155 | A G T C G T A C A G C T C T A G A G T C C G A T A C T G C G T A A C T G G T C A | Zic(Zf)/Cerebellum-ZIC1.2-ChIP-Seq(GSE60731)/Homer | 1e-4 | -1.066e+01 | 0.0001 | 749.0 | 10.64% | 3876.9 | 9.21% | motif file (matrix) | svg |
| 156 | C A T G T G A C C G T A A G T C T A C G G C A T A C T G G T C A A T G C A G T C | bHLHE41(bHLH)/proB-Bhlhe41-ChIP-Seq(GSE93764)/Homer | 1e-4 | -9.938e+00 | 0.0001 | 938.0 | 13.33% | 4968.6 | 11.80% | motif file (matrix) | svg |
| 157 | G C A T C G T A C G A T A C T G A G T C G C T A C T G A C G T A C A G T A C T G C G T A T C A G | Oct6(POU,Homeobox)/NPC-Pou3f1-ChIP-Seq(GSE35496)/Homer | 1e-4 | -9.595e+00 | 0.0002 | 731.0 | 10.39% | 3811.5 | 9.05% | motif file (matrix) | svg |
| 158 | A G T C G A C T C A G T G T A C A G T C A T C G T C A G A C T G G T C A C G T A | Stat3(Stat)/mES-Stat3-ChIP-Seq(GSE11431)/Homer | 1e-4 | -9.581e+00 | 0.0002 | 486.0 | 6.91% | 2446.7 | 5.81% | motif file (matrix) | svg |
| 159 | G A C T C G T A A C G T A C T G A G T C C G T A C T G A C G T A C A G T A C G T G T C A T C A G | Brn1(POU,Homeobox)/NPC-Brn1-ChIP-Seq(GSE35496)/Homer | 1e-4 | -9.341e+00 | 0.0002 | 544.0 | 7.73% | 2774.4 | 6.59% | motif file (matrix) | svg |
| 160 | C A T G G A C T G C A T A C T G A G C T A C T G A C T G C G T A G C A T A G C T A T C G T A C G | Foxh1(Forkhead)/hESC-FOXH1-ChIP-Seq(GSE29422)/Homer | 1e-4 | -9.270e+00 | 0.0003 | 834.0 | 11.85% | 4404.4 | 10.46% | motif file (matrix) | svg |
| 161 | C G A T A C G T A C G T A C G T C G T A A G C T C A G T C T A G A T C G A C T G | HOXB13(Homeobox)/ProstateTumor-HOXB13-ChIP-Seq(GSE56288)/Homer | 1e-3 | -9.160e+00 | 0.0003 | 1697.0 | 24.11% | 9371.2 | 22.25% | motif file (matrix) | svg |
| 162 | C T G A A G C T A C G T A C G T A G T C G A C T G A C T C T G A C T G A C T A G C G T A C G T A | STAT6(Stat)/CD4-Stat6-ChIP-Seq(GSE22104)/Homer | 1e-3 | -9.128e+00 | 0.0003 | 708.0 | 10.06% | 3698.8 | 8.78% | motif file (matrix) | svg |
| 163 | G T A C G C A T C T G A C G T A G A C T A G C T C A T G T G C A C T G A A C G T G A C T C G T A | Prop1(Homeobox)/GHFT1-PROP1.biotin-ChIP-Seq(GSE77302)/Homer | 1e-3 | -9.111e+00 | 0.0003 | 1245.0 | 17.69% | 6758.2 | 16.05% | motif file (matrix) | svg |
| 164 | T A C G A T C G T A G C G A T C A C T G A C G T A G T C A C G T C T A G A T C G | Smad4(MAD)/ESC-SMAD4-ChIP-Seq(GSE29422)/Homer | 1e-3 | -8.823e+00 | 0.0004 | 1474.0 | 20.94% | 8094.6 | 19.22% | motif file (matrix) | svg |
| 165 | A T G C T C G A A G T C A G C T A C G T G T A C A G T C G C T A C T A G C A T G G T C A C T G A T C A G A G T C | Stat3+il21(Stat)/CD4-Stat3-ChIP-Seq(GSE19198)/Homer | 1e-3 | -8.578e+00 | 0.0005 | 747.0 | 10.61% | 3938.8 | 9.35% | motif file (matrix) | svg |
| 166 | T A G C G A T C G T A C C T G A G C A T C T G A C G T A T G C A C G T A G A T C | Hoxa13(Homeobox)/ChickenMSG-Hoxa13.Flag-ChIP-Seq(GSE86088)/Homer | 1e-3 | -8.514e+00 | 0.0005 | 3765.0 | 53.50% | 21639.6 | 51.38% | motif file (matrix) | svg |
| 167 | A C G T C T A G A G C T A C G T A C G T C T G A A G T C G A C T A G C T C G T A | FOXM1(Forkhead)/MCF7-FOXM1-ChIP-Seq(GSE72977)/Homer | 1e-3 | -8.321e+00 | 0.0006 | 1875.0 | 26.64% | 10455.7 | 24.83% | motif file (matrix) | svg |
| 168 | T G A C C T A G A C T G T A G C C G A T A C T G A T G C C A T G A T C G A T C G A T C G T A G C C T G A T A G C G C T A A C T G C G T A A G C T C G T A C T G A | GATA:SCL(Zf,bHLH)/Ter119-SCL-ChIP-Seq(GSE18720)/Homer | 1e-3 | -8.070e+00 | 0.0008 | 145.0 | 2.06% | 644.9 | 1.53% | motif file (matrix) | svg |
| 169 | G A C T C T A G C T A G C T A G A C T G T C G A C T G A C T A G C T A G C T A G G T A C G T C A | ZNF467(Zf)/HEK293-ZNF467.GFP-ChIP-Seq(GSE58341)/Homer | 1e-3 | -8.031e+00 | 0.0008 | 620.0 | 8.81% | 3242.5 | 7.70% | motif file (matrix) | svg |
| 170 | T C G A T C G A T A G C G T A C T C A G T A C G C G T A C G T A T C A G A G C T | GABPA(ETS)/Jurkat-GABPa-ChIP-Seq(GSE17954)/Homer | 1e-3 | -7.862e+00 | 0.0010 | 780.0 | 11.08% | 4153.5 | 9.86% | motif file (matrix) | svg |
| 171 | T C A G T A G C G A C T C A T G C T G A A T C G G C A T G T A C C G T A A C T G T A G C T G C A | MafK(bZIP)/C2C12-MafK-ChIP-Seq(GSE36030)/Homer | 1e-3 | -7.770e+00 | 0.0011 | 279.0 | 3.96% | 1361.6 | 3.23% | motif file (matrix) | svg |
| 172 | T A C G A T C G G A T C G T C A C T G A G C A T C G A T G C T A T C G A G C T A | Unknown(Homeobox)/Limb-p300-ChIP-Seq/Homer | 1e-3 | -7.730e+00 | 0.0011 | 1100.0 | 15.63% | 5989.8 | 14.22% | motif file (matrix) | svg |
| 173 | G A C T C T A G C T A G A G T C T G C A A C T G A C G T A C G T C T A G T C A G | AMYB(HTH)/Testes-AMYB-ChIP-Seq(GSE44588)/Homer | 1e-3 | -7.600e+00 | 0.0013 | 1674.0 | 23.79% | 9323.0 | 22.14% | motif file (matrix) | svg |
| 174 | A C T G A C G T C A T G A T C G A T C G T G A C A C T G A T C G A T C G T G C A C T G A C G T A | E2F3(E2F)/MEF-E2F3-ChIP-Seq(GSE71376)/Homer | 1e-3 | -7.377e+00 | 0.0016 | 249.0 | 3.54% | 1208.0 | 2.87% | motif file (matrix) | svg |
| 175 | T A G C G T A C C G T A C T A G A C T G T G C A C G T A A T G C C G T A A T C G | AR-halfsite(NR)/LNCaP-AR-ChIP-Seq(GSE27824)/Homer | 1e-3 | -7.356e+00 | 0.0016 | 3741.0 | 53.15% | 21575.4 | 51.23% | motif file (matrix) | svg |
| 176 | C T G A A C G T A C G T A C G T A G T C G A C T C G A T C T G A A C T G C G T A C G T A T C G A | STAT5(Stat)/mCD4+-Stat5-ChIP-Seq(GSE12346)/Homer | 1e-3 | -7.323e+00 | 0.0016 | 446.0 | 6.34% | 2292.3 | 5.44% | motif file (matrix) | svg |
| 177 | A T G C C T G A G A C T A C G T A C G T G T A C G A T C C G A T C T A G C A T G C G T A C G T A C T G A G A C T | STAT1(Stat)/HelaS3-STAT1-ChIP-Seq(GSE12782)/Homer | 1e-3 | -7.293e+00 | 0.0017 | 388.0 | 5.51% | 1971.9 | 4.68% | motif file (matrix) | svg |
| 178 | A C T G T C A G A G C T G A C T C A T G A G T C A G T C G C T A C G A T C T A G T C A G G T A C C T G A T C G A | Rfx1(HTH)/NPC-H3K4me1-ChIP-Seq(GSE16256)/Homer | 1e-3 | -7.276e+00 | 0.0017 | 190.0 | 2.70% | 894.4 | 2.12% | motif file (matrix) | svg |
| 179 | A T G C G A C T A C T G C A G T G A T C A C G T T A C G T A C G | Smad2(MAD)/ES-SMAD2-ChIP-Seq(GSE29422)/Homer | 1e-3 | -7.261e+00 | 0.0017 | 1440.0 | 20.46% | 7980.9 | 18.95% | motif file (matrix) | svg |
| 180 | G A T C G C A T C T G A C G T A A G C T A G C T C G T A T C G A C T G A A C G T G C A T C G T A | Phox2a(Homeobox)/Neuron-Phox2a-ChIP-Seq(GSE31456)/Homer | 1e-3 | -7.107e+00 | 0.0020 | 772.0 | 10.97% | 4139.2 | 9.83% | motif file (matrix) | svg |
| 181 | T A C G C T G A T C G A C G A T C T A G C T A G T C G A C T G A T C G A T C G A C G T A T C G A G C A T C A T G C G T A T A C G G C A T T G A C C G T A A G C T | NFAT:AP1(RHD,bZIP)/Jurkat-NFATC1-ChIP-Seq(Jolma\_et\_al.)/Homer | 1e-3 | -6.940e+00 | 0.0024 | 221.0 | 3.14% | 1068.0 | 2.54% | motif file (matrix) | svg |
| 182 | T G A C T A G C T C A G T C G A T C G A C G T A A G T C C G T A C G T A C G A T C T A G T A C G | Sox7(HMG)/ESC-Sox7-ChIP-Seq(GSE133899)/Homer | 1e-2 | -6.894e+00 | 0.0025 | 362.0 | 5.14% | 1839.8 | 4.37% | motif file (matrix) | svg |
| 183 | A T G C G A T C G A C T A G C T C G A T C G A T G T C A C G A T T C G A A T C G T A G C T A G C | TATA-Box(TBP)/Promoter/Homer | 1e-2 | -6.893e+00 | 0.0025 | 1795.0 | 25.50% | 10073.6 | 23.92% | motif file (matrix) | svg |
| 184 | C T G A A T G C C G T A A C G T A G T C A G T C A C G T A C T G A T C G G C A T | SPDEF(ETS)/VCaP-SPDEF-ChIP-Seq(SRA014231)/Homer | 1e-2 | -6.855e+00 | 0.0025 | 1035.0 | 14.71% | 5658.6 | 13.44% | motif file (matrix) | svg |
| 185 | T C G A G C A T A T G C C T G A A T G C T A G C A G T C G T A C T C G A A G C T | Srebp1a(bHLH)/HepG2-Srebp1a-ChIP-Seq(GSE31477)/Homer | 1e-2 | -6.791e+00 | 0.0027 | 175.0 | 2.49% | 824.9 | 1.96% | motif file (matrix) | svg |
| 186 | T G A C G T C A A G T C G C T A C T A G A G T C C T G A C A T G A C T G C T A G T A C G T C A G | Zic2(Zf)/ESC-Zic2-ChIP-Seq(SRP197560)/Homer | 1e-2 | -6.787e+00 | 0.0027 | 297.0 | 4.22% | 1484.4 | 3.52% | motif file (matrix) | svg |
| 187 | C G T A A C G T A G C T C G A T C T A G G T A C C G T A A G C T C G T A G C T A | Oct4(POU,Homeobox)/mES-Oct4-ChIP-Seq(GSE11431)/Homer | 1e-2 | -6.527e+00 | 0.0034 | 806.0 | 11.45% | 4359.4 | 10.35% | motif file (matrix) | svg |
| 188 | G A T C C T G A A G T C C G A T C G A T G A T C A G T C A C T G A T C G A G C T | Elk4(ETS)/Hela-Elk4-ChIP-Seq(GSE31477)/Homer | 1e-2 | -6.488e+00 | 0.0036 | 383.0 | 5.44% | 1968.2 | 4.67% | motif file (matrix) | svg |
| 189 | C G A T A C G T C G T A C G T A A C G T A G C T T G C A C T G A C G T A A C G T A C G T C G T A | Phox2b(Homeobox)/CLBGA-PHOX2B-ChIP-Seq(GSE90683)/Homer | 1e-2 | -6.305e+00 | 0.0043 | 426.0 | 6.05% | 2214.4 | 5.26% | motif file (matrix) | svg |
| 190 | T C A G T A C G G A T C G T A C T C G A C G A T C T G A C G T A G C T A C G T A | Hoxd11(Homeobox)/ChickenMSG-Hoxd11.Flag-ChIP-Seq(GSE86088)/Homer | 1e-2 | -6.247e+00 | 0.0045 | 3424.0 | 48.65% | 19762.5 | 46.92% | motif file (matrix) | svg |
| 191 | A C T G A G T C G T C A C G T A A G T C C G T A C T A G C T A G G A C T C A T G | SCRT1(Zf)/HEK293-SCRT1.eGFP-ChIP-Seq(Encode)/Homer | 1e-2 | -6.227e+00 | 0.0045 | 296.0 | 4.21% | 1494.0 | 3.55% | motif file (matrix) | svg |
| 192 | C T A G T A C G G A T C G T C A T G C A A C G T T G C A G C T A T C G A T G C A | Hoxa9(Homeobox)/ChickenMSG-Hoxa9.Flag-ChIP-Seq(GSE86088)/Homer | 1e-2 | -6.083e+00 | 0.0052 | 3560.0 | 50.58% | 20588.7 | 48.89% | motif file (matrix) | svg |
| 193 | T G A C C T G A C T A G C T G A C G T A A G T C C T G A A C G T G C A T T A G C G C A T A T C G G A C T G A C T G A T C | GRE(NR),IR3/RAW264.7-GRE-ChIP-Seq(Unpublished)/Homer | 1e-2 | -5.992e+00 | 0.0057 | 269.0 | 3.82% | 1352.4 | 3.21% | motif file (matrix) | svg |
| 194 | A T G C G A T C C G A T A C G T A C G T A C T G C A G T A G C T | Sox3(HMG)/NPC-Sox3-ChIP-Seq(GSE33059)/Homer | 1e-2 | -5.659e+00 | 0.0079 | 2129.0 | 30.25% | 12121.2 | 28.78% | motif file (matrix) | svg |
| 195 | T A C G T C A G G A T C G T A C T C G A G A C T G C T A G C T A G C T A C G T A G A T C G T C A | CDX4(Homeobox)/ZebrafishEmbryos-Cdx4.Myc-ChIP-Seq(GSE48254)/Homer | 1e-2 | -5.639e+00 | 0.0080 | 1466.0 | 20.83% | 8230.4 | 19.54% | motif file (matrix) | svg |
| 196 | G C T A G C A T G A C T G C A T T C A G G T A C G C T A G C A T C T G A G C T A T A G C G C T A C T G A C G A T C T A G | OCT4-SOX2-TCF-NANOG(POU,Homeobox,HMG)/mES-Oct4-ChIP-Seq(GSE11431)/Homer | 1e-2 | -5.409e+00 | 0.0100 | 371.0 | 5.27% | 1937.3 | 4.60% | motif file (matrix) | svg |
| 197 | G C T A A G T C T A C G T G C A A T C G T C A G G C T A T C G A T C A G A G C T | ELF5(ETS)/T47D-ELF5-ChIP-Seq(GSE30407)/Homer | 1e-2 | -5.235e+00 | 0.0119 | 760.0 | 10.80% | 4158.6 | 9.87% | motif file (matrix) | svg |
| 198 | C G A T C T A G C T G A A T G C C T G A T C G A C G T A C T G A T C G A T A G C A G T C C G T A A C T G T C G A A T G C | Hand2(bHLH)/Mesoderm-Hand2-ChIP-Seq(GSE61475)/Homer | 1e-2 | -5.211e+00 | 0.0121 | 487.0 | 6.92% | 2599.5 | 6.17% | motif file (matrix) | svg |
| 199 | T G C A C T G A A T G C G T C A A C T G A C T G C G T A C G T A C T A G A G C T | Ets1-distal(ETS)/CD4+-PolII-ChIP-Seq(Barski\_et\_al.)/Homer | 1e-2 | -5.163e+00 | 0.0127 | 320.0 | 4.55% | 1660.8 | 3.94% | motif file (matrix) | svg |
| 200 | A G T C A T C G G C A T C T A G A C T G T A C G C G A T T C A G C A T G A G C T T A G C G A T C | GLI3(Zf)/Limb-GLI3-ChIP-Chip(GSE11077)/Homer | 1e-2 | -4.970e+00 | 0.0153 | 82.0 | 1.17% | 368.4 | 0.87% | motif file (matrix) | svg |
| 201 | C G T A C T A G T C A G T C A G A G T C A T G C A G T C G C A T A G C T A C G T A T C G C G A T | Sox9(HMG)/Limb-SOX9-ChIP-Seq(GSE73225)/Homer | 1e-2 | -4.898e+00 | 0.0163 | 973.0 | 13.82% | 5408.0 | 12.84% | motif file (matrix) | svg |
| 202 | A C T G G A C T A G T C C T G A G A T C T C A G A T G C G A C T A G T C A T G C T A G C A G C T A T C G T G C A | PAX5(Paired,Homeobox),condensed/GM12878-PAX5-ChIP-Seq(GSE32465)/Homer | 1e-2 | -4.724e+00 | 0.0194 | 82.0 | 1.17% | 372.8 | 0.89% | motif file (matrix) | svg |
| 203 | T A C G T A C G G T A C A T C G A C T G T A C G T C G A C T G A T C G A A T C G | E2F6(E2F)/Hela-E2F6-ChIP-Seq(GSE31477)/Homer | 1e-2 | -4.676e+00 | 0.0202 | 192.0 | 2.73% | 965.1 | 2.29% | motif file (matrix) | svg |
